# Genome-based selection and application of food-grade microbes for chickpea milk fermentation towards increased l-lysine content, elimination of indigestible sugars, and improved flavour

**DOI:** 10.1186/s12934-021-01595-2

**Published:** 2021-05-28

**Authors:** Muzi Tangyu, Michel Fritz, Rosa Aragao-Börner, Lijuan Ye, Biljana Bogicevic, Christoph J. Bolten, Christoph Wittmann

**Affiliations:** 1 Institute of Systems Biotechnology, Saarland University, Saarbrücken, Germany; 2 Nestlé Research Center, Lausanne, Switzerland; 3 Nestlé Product Technology Center Food, Singen, Germany

**Keywords:** l-lysine, Lactic acid bacteria, *Lacticaseibacillus paracasei*, *Bacillus amyloliquefaciens*, ^13^C isotope study, Chickpea, Plant-based milk alternative, Plant milk, fermentation, Flavour, Indigestible sugar, Raffinose, Stachyose

## Abstract

**Background:**

Plant-based milk alternatives are more popular than ever, and chickpea-based milks are among the most commercially relevant products. Unfortunately, limited nutritional value because of low levels of the essential amino acid l-lysine, low digestibility and unpleasant taste are challenges that must be addressed to improve product quality and meet consumer expectations.

**Results:**

Using *in-silico* screening and food safety classifications, 31 strains were selected as potential l-lysine producers from approximately 2,500 potential candidates. Beneficially, 30% of the isolates significantly accumulated amino acids (up to 1.4 mM) during chickpea milk fermentation, increasing the natural level by up to 43%. The best-performing strains, *B. amyloliquefaciens* NCC 156 and *L. paracasei* subsp*. paracasei* NCC 2511, were tested further. De novo lysine biosynthesis was demonstrated in both strains by ^13^C metabolic pathway analysis. Spiking small amounts of citrate into the fermentation significantly activated l-lysine biosynthesis in NCC 156 and stimulated growth. Both microbes revealed additional benefits in eliminating indigestible sugars such as stachyose and raffinose and converting off-flavour aldehydes into the corresponding alcohols and acids with fruity and sweet notes.

**Conclusions:**

*B. amyloliquefaciens* NCC 156 and *L. paracasei* subsp*. paracasei* NCC 2511 emerged as multi-benefit microbes for chickpea milk fermentation with strong potential for industrial processing of the plant material. Given the high number of l-lysine-producing isolates identified in silico, this concept appears promising to support strain selection for food fermentation.

**Supplementary Information:**

The online version contains supplementary material available at 10.1186/s12934-021-01595-2.

## Background

Plant-based milk alternatives are growing in popularity. They offer attractive properties for consumers by being ecologically more sustainable and animal-welfare-friendly than dairy milks, lactose-free, and vegan. Notably, the world market for such products is continuously increasing and is expected to surpass US$ 26 billion by 2023 [1]. As prominent example, chickpea (*Cicer arietinum L.)*, one of the oldest and most widely consumed legumes worldwide [2], is regarded an attractive source of milk-alternative consumer products [3, 4] with good protein quality [5, 6].

Unfortunately, suspensions of chickpea flour (termed chickpea milk below due to their milk-like appearance) do not match animal milk in all desired characteristics, which is a limitation that is generally observed for plant-based milk alternatives [7]. This limitation also holds for the essential amino acid l-lysine, which is required for hormone formation, catalytic and structural proteins, and immune system support and is therefore one of the most impacting nutrients [8]. Critically, it exhibits much lower abundance in chickpea-based milk than in animal milk, approximately half of the amount in a typical 10% dry matter formulation [9, 10]). Moreover, chickpea milk contains elevated levels of indigestible sugars such as raffinose and stachyose, which cause flatulence, diarrhoea, and other discomfort upon consumption [11]. In addition, it exhibits a grassy and beany taste that does not meet consumer expectations [12].

Microbial fermentation is a well-established way to improve the nutritional quality of plant-based milk alternatives [1]. For example, fermentation of milks from peanut, soy, cowpea, and mung bean increased protein quality and digestibility and occasionally also the l-lysine content [13–19]. Admittedly, only selected microbial strains improve desired features [20]. For example, natural microbes rarely overproduce l-lysine [21].

**Fig. 1:**
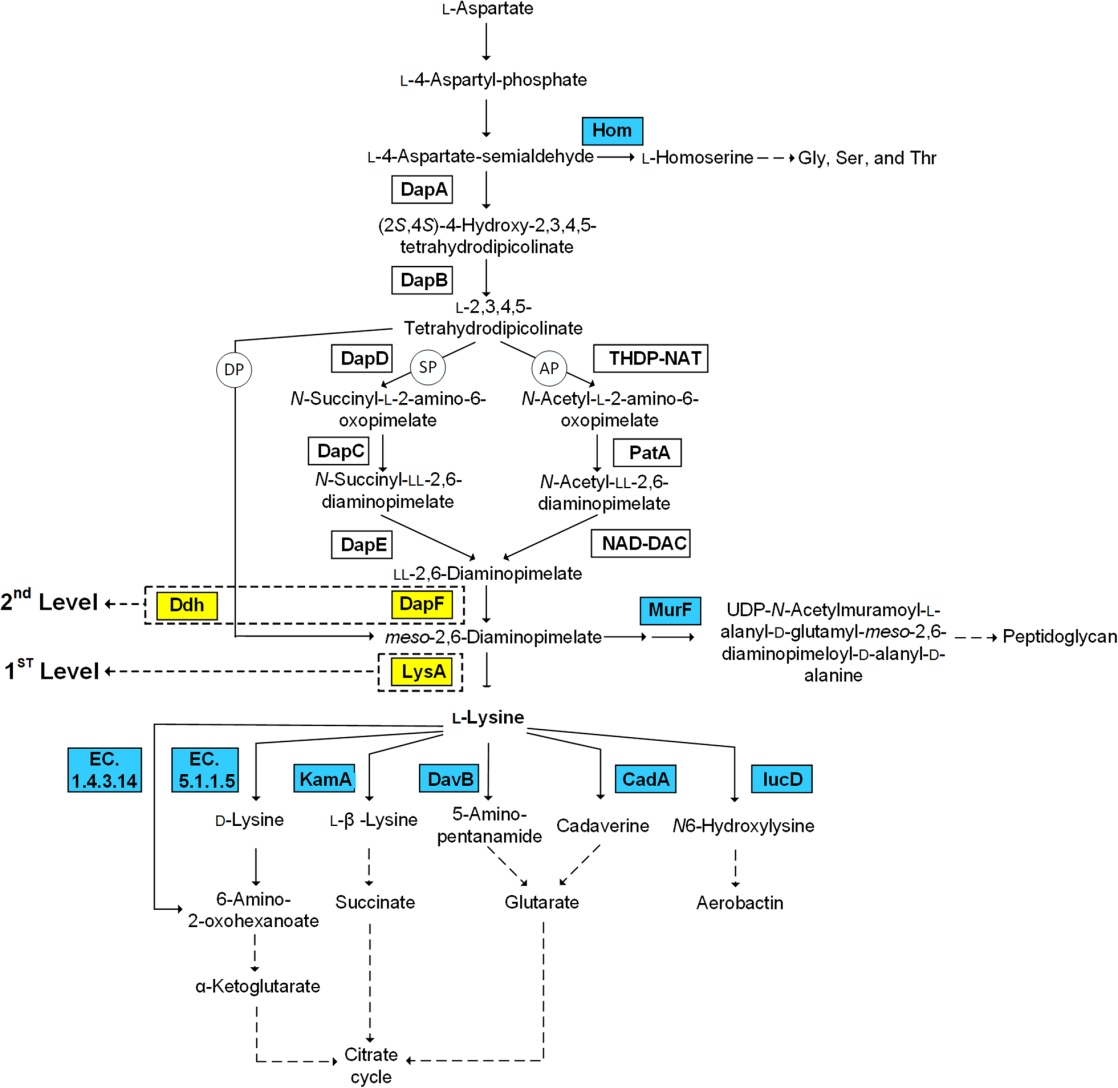
Microbial pathways for the synthesis and degradation of l-lysine. Potential l-lysine overproducers were identified from the NCC database based on the presence and absence of key genes related to the well-established routes of l-lysine metabolism [95]. Enzymes shown in yellow were essential for l-lysine biosynthesis, whereas enzymes shown in blue were involved in competing pathways and l-lysine degradation [96–98]. The presence of diaminopimelate decarboxylase (*lysA*) was regarded as essential for potential l-lysine-producing strains (level 1). Diaminopimelate epimerase (*dapF*) and diaminopimelate dehydrogenase (*ddh*) were then used to infer the putative pathway involved (level 2). *DP* dehydrogenase pathway, *SP* succinylase pathway, *AP* acetylase pathway, *DapA* 4-hydroxy-tetrahydrodipicolinate synthase, *DapB* 4-hydroxytetrahydrodipicolinate reductase, *DapD* tetrahydrodipicolinate succinylase, *DapC* succinyl-amino-ketopimelate transaminase, *DapE* *N*-succinyl-diaminopimelate desuccinylase, *DapF* diaminopimelate epimerase, *Ddh* diaminopimelate dehydrogenase, *THDP-NAT* tetrahydrodipicolinate acetylase, *PatA* *N*-acetyl-amino-ketopimelate aminotransferase, *NAD-DAC* *N*-acetyl-diaminopimelate deacetylase, *LysA* diaminopimelate decarboxylase, *Hom* homoserine dehydrogenase, *MurF* UDP-*N*-acetylmuramoylalanyl-d-glutamyl-2,6-diamino-pimelate-d-alanyl-d-alanyl ligase, *EC 1.4.3.14* l-lysine oxidase, *EC 5.1.1.5* lysine racemase, *KamA* l-lysine 2,3-aminomutase, *DavB* lysine 2-monooxygenase; CadA, lysine decarboxylase; LucD, lysine N6-hydroxylase

Here, we present a systematic workflow to fortify a chickpea milk using fermentation with natural food-grade microbes. To improve upon the low level of l-lysine, more than 30 strains were selected as candidates based on their genomic repertoire related to l-lysine metabolism (Fig. 1). A screening round revealed that one-third (9) of the tested strains significantly accumulated l-lysine during chickpea milk fermentation. ^13^C isotope studies showed that the two best-performing strains, *Lacticaseibacillus paracasei* subsp*. paracasei* Nestlé Culture Collection (NCC) 2511 and *Bacillus amyloliquefaciens* NCC 156, synthetize l-lysine de novo. Both microbes were used for a more detailed investigation of the fermentation process. They beneficially altered the amino acid and protein profile, utilized (indigestible) carbohydrates, and formed desired flavour molecules, which highlights them as microbes well suited for chickpea milk fermentation.

## Results

### Genome-based selection of food-grade microbes for l-lysine production

Bacterial genomes of the NCC were analysed for their features related to l-lysine, including l-lysine biosynthesis, l-lysine degradation, and pathways competing with l-lysine biosynthesis for carbon precursors (Fig. 1). A component from the pathway architecture for bacterial l-lysine biosynthesis, the *lysA* gene (encoding diaminopimelate decarboxylase) was inferred as a premium selection criterion to identify potential l-lysine producers. This enzyme catalyses the terminal step of l-lysine synthesis, downstream of *meso*-diaminopimelate, where all synthetic routes converge (Fig. 1). Therefore, the presence of *lysA* was an essential (minimal) feature required for l-lysine formation. In total, 2472 bacterial NCC genomes contained an annotated *lysA* gene, and most of them belonged to the orders *Lactobacillales*, *Bacillales*, *Bifidobacteriales*, and *Corynebacteriales*. Fewer than half of the genomes (1,117) additionally included a *dapF* gene, potentially indicating the succinylase and/or acetylase route to synthesize l-lysine (Fig. 1). Only 14 strains contained the *ddh* gene (encoding diaminopimelate dehydrogenase) as a key step of the dehydrogenase pathway.

The selection was narrowed down by considering only strains approved for use in the food and feed chain according to the European Food Safety Authority [22]. Among all potential l-lysine producers, i. e. all strains that contained *lys*A plus either *dap*F or *ddh*, 945 strains exhibited the qualified presumption of safety (QPS) status and therefore fulfilled this extra requirement. They belonged to five different families (*Lactobacillaceae, Bacillaceae, Bifidobacteriaceae, Corynebacteraceae, Carnobacteriaceae*), including 12 genera and 33 different species. None of the strains that contained a *ddh* gene met the food safety constraint. *Carnobacterium* and *Pediococcus* were not further considered because they are mainly associated with producing bacteriocins for food preservation [23, 24], and the fermentation of materials other than milk such as wine, cheese, sausage, and cabbage [25], leaving *Lactobacillaceae* (757), *Bacillaceae* (34), *Bifidobacteriaceae* (119), and *Corynebacteraceae* (2) for the final selection. Accordingly, 31 potential producers (all containing *lysA* and *dapF* that covered the identified taxonomic diversity were selected for experimental studies. The selected strains represented 10 genera and 19 different species (Additional file 1: Table S1): (i) *Lactobacillus helveticus* (3), *Lactobacillus johnsonii* (3)*, Lactobacillus acidophilus* (1)*, Lactobacillus delbrueckii* (1)*, Limosilactobacillus reuteri* (3)*, Limosilactobacillus pontis* (1), *Lacticaseibacillus paracasei* (2)*, Lactiplantibacillus plantarum* (1), *Fructilactobacillus sanfranciscensis* (3), *Levilactobacillus brevis* (1)*, Lentilactobacillus hilgardii* (1); (ii) *Bifidobacterium longum* (1) and *Bifidobacterium infantis* (1)*;* (iii) *Bacillus amyloliquefaciens* (2)*, Bacillus flexus* (2)*, Bacillus licheniformis* (1)*, Bacillus pumilus* (1), and *Bacillus subtilis* (1)*;* and (iv) *Corynebacterium stationis* (2)*.* In addition, *Lactobacillus jensenii* NCC 2867, lacking *lysA*, was used as a negative control. Regarding oxygen sensitivity, the selected strains were classified as obligate anaerobes (6), aerotolerant (17), and obligate aerobes (9).

### Nutritional characteristics of chickpea milk

Initial tests revealed that a one-step pre-treatment was not suitable to provide homogeneous, sterile chickpea milk for fermentation. Standard autoclaving (121 °C, 15 min) resulted in undesired starch gelation and an inhomogeneous suspension, while pasteurization (63 °C, 5 h) failed to avoid contamination in the subsequent fermentation studies (Additional file 1: Fig. S1). After several tests, a two-step heat pre-treatment was developed. It consisted of an initial phase of heating and stirring (75 °C, 2 h), followed by autoclaving (121 °C, 15 min). Notably, this treatment fully avoided starch gelation. Furthermore, it provided a sterile, homogenous, and easy to process suspension (Additional file 1: Fig. S1).

**Fig. 2:**
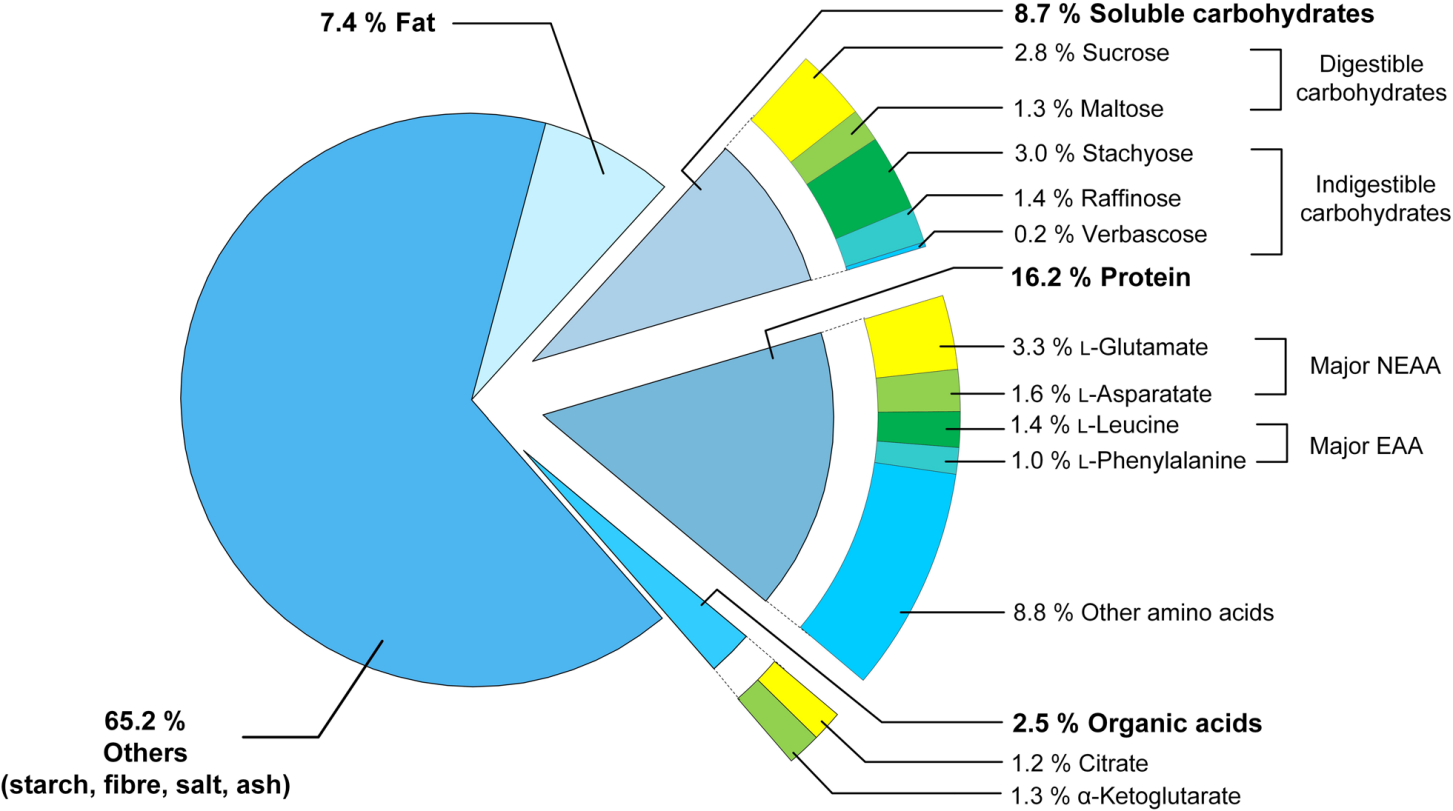
Composition of non-filtered chickpea milk used for the fermentation studies. All values are related to the dry mass (w/w). n = 3

From a chemical composition viewpoint, the unfiltered chickpea milk contained 16.2% (w/w, dry mass) protein, 8.7% soluble carbohydrates, 7.4% fat, 2.5% organic acid, and a remaining fraction accounting for 65.2% (e.g., starch, fibres, ash, and salt) (Fig. 2) which well compared to previous studies, given seasonal and geographical fluctuations [3, 6, 9, 26, 27]. Among the nonessential amino acids, l-glutamate (3.3%) and l-aspartate (1.6%) were most abundant. The major essential amino acids were l-leucine (1.4%) and l-phenylalanine (1.0%). As expected, the level of l-lysine (8.7 mg (g dry mass)^−1^) was only 30–70% of the value observed for animal milk [28]. Soluble carbohydrates comprised digestible (sucrose, maltose) and indigestible carbohydrates (raffinose, stachyose, verbascose). Stachyose (3.0%, w/w, dry mass), sucrose (2.8%), raffinose (1.4%), and maltose (1.2%) were the major soluble sugars. Verbascose was present in lower amounts, whereas the levels of glucose and fructose were negligible. In addition, chickpea milk contained organic acids such as 1.3% α-ketoglutarate (w/w, dry mass), 1.2% citrate, and trace amounts of succinate.

### Evaluation of the pre-selected microbes for chickpea milk fermentation

**Fig. 3:**
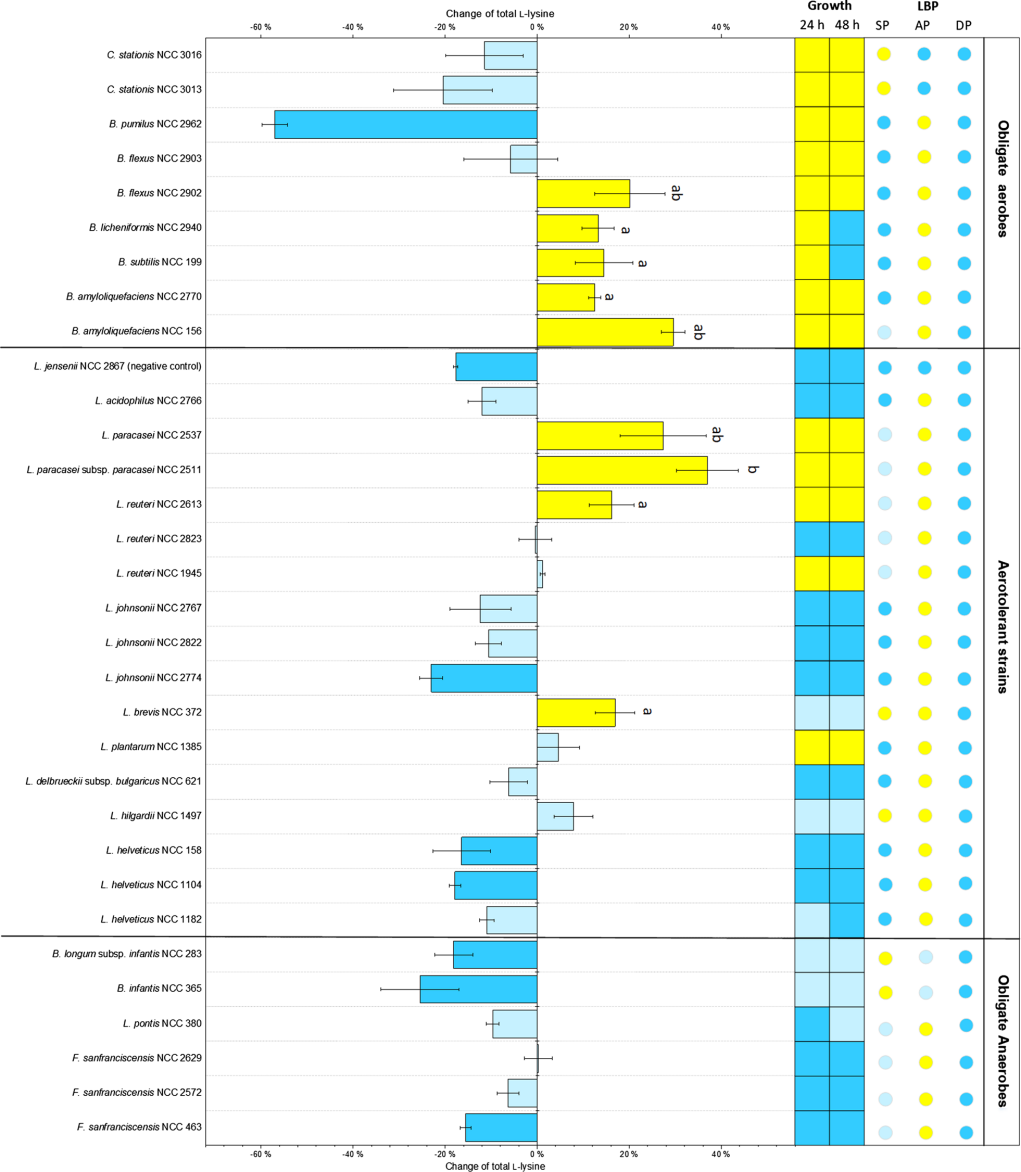
Growth and l-lysine production of pre-selected food-grade strains during fermentation of chickpea milk. *Lactobacillus jensenii* was included as a negative control and is correspondingly annotated. The l-lysine concentration after 48 h of fermentation was significantly increased (yellow, p < 0.05), significantly decreased (blue, p < 0.05), or not significantly changed (light blue). The coloured squares represent the change in cfu after 24 h and 48 h: strong growth (yellow, increase > 1 log cfu mL^−1^), weak growth (light blue, increase < 1 log cfu mL^−1^), and no growth (blue, no change). In addition, the genomic repertoire of the strains for l-lysine biosynthesis is shown (Fig. 1): yellow, completely annotated pathway; light blue, partially annotated pathway; and blue, pathway not annotated. *SP* succinylase pathway, *AP* acetylase pathway, *DP* dehydrogenase pathway. n = 3

The selected 31 strains differed in their genomic repertoire regarding the number of completely and partially annotated l-lysine biosynthetic pathways (Fig. 3). They were next experimentally evaluated for their capability to ferment chickpea milk, and *L. jensenii* was included as a negative control (Fig. 3). Generally, the established fermentation process was highly reproducible, which enabled a clear evaluation of strain performance. In total, 60% of the strains (19 out of 32) were able to grow (Additional file 1: Table S2). Depending on species and strain, the observed increase in the number of living cells ranged from 0.2 to 3.2 log cfu mL^−1^. Generally, obligate aerobic and aerotolerant strains grew well, while obligate anaerobes showed weak or even no growth. The bacilli and corynebacteria showed the strongest growth. The cfu numbers for these strains increased at least tenfold (1 log) while the bifidobacteria grew only weakly. For the lactobacilli, the outcome was mixed. Some strains grew well (*L. plantarum, L. paracasei* subsp*. paracasei* and two out of three *L. reuteri*), while others (*F. sanfranciscensis, L. helveticus, L. johnsonii, L. acidophilus*, the remaining *L. reuteri* and the negative control strain *L. jensenii*) did not grow.

Notably, 30% of the strains (9 out of 31 potential producers) increased the level of l-lysine. The producers were exclusively found among four genera: *Lacticaseibacillus, Limosilactobacillus, Levilactobacillus,* and *Bacillus*. High and low producers were equally distributed between lactic acid bacteria and *Bacillus* groups. The best, third best, fifth best, and sixth best producers were lactic acid bacteria strains (*L. paracasei* subsp*. paracasei* NCC 2511, *L. paracasei* NCC 2537, *L. reuteri* NCC 2613 and *L. brevis* NCC 372), while the second, fourth, seventh, eighth and ninth highest l-lysine levels were achieved by *Bacillus* strains (*B. amyloliquefaciens* NCC 156, *B. flexus* NCC 2902, *B. flexus* NCC 2940, *B. subtilis* NCC 199, *B. licheniformis* NCC 2940, *B. amyloliquefaciens* NCC 2770). *L. paracasei* subsp*. paracasei* NCC 2511 and *B. amyloliquefaciens* NCC 156 increased the l-lysine content by 37% and 30%, respectively. In contrast, none of the *Bifidobacterium* and *Corynebacterium* isolates increased the l-lysine level. Several isolates degraded l-lysine. *B. pumilus* NCC 2962 decreased the l-lysine content by over 50%. Fifteen strains did not alter the content significantly.

*L. paracasei* subsp*. paracasei* NCC 2511 and *B. amyloliquefaciens* NCC 156 appeared most promising for further studies regarding their superior capability to overproduce l-lysine. The inspection of their genomes revealed that they possessed a rich repertoire to potentially improve also other important traits of the plant milk. They contained several genes, functionally assigned to catalyse flavour formation, such as alcohol dehydrogenase [29], aldehyde dehydrogenase [29], and enzymes of branched chain amino acid metabolism [30] (Additional file 1: Table S3). Moreover, genes encoding carbohydrate degrading enzymes, known to be involved in the removal of indigestible sugars [31], were present. Therefore, both strains were subjected to a more-detailed characterization.

### Fermentation of chickpea milk using *B. amyloliquefaciens* NCC 156

**Fig. 4:**
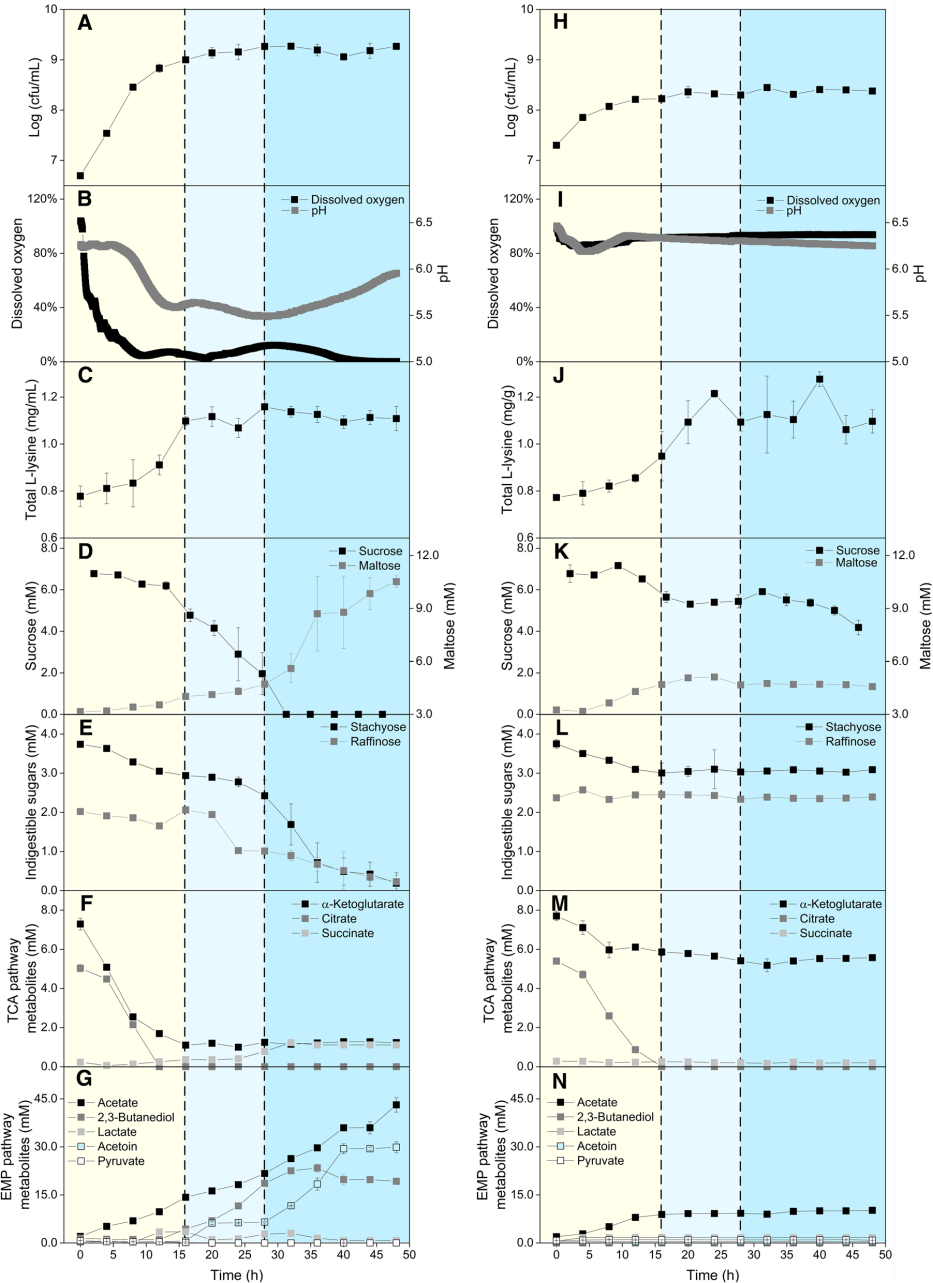
Time-resolved fermentation of chickpea milk using *B. amyloliquefaciens* NCC 156 (left, **A**–**G**) and *L. paracasei* subsp*. paracasei* NCC 2511 (right, **H**–**N**). The data comprise living cell number (cfu) (**A**, **H**), dissolved oxygen level and pH value (B, I), and the concentrations of total l-lysine (**C**, **J**), sucrose, maltose (**D**, **K**), raffinose, stachyose (**E**, **L**), citrate, α-ketoglutarate, succinate (**F**, **M**), acetate, acetoin, 2,3-butanediol, lactate, and pyruvate (**G**, **N**). n = 3

The physiology of *B. amyloliquefaciens* NCC 156 during chickpea milk fermentation was monitored over a period of 48 h (Fig. 4). The microbe went through several distinct phases of an obviously altered metabolism. The initial phase was the main phase of growth. The cells immediately started to proliferate, and the cfu number increased almost 1000-fold during the first 8 h. Notably, the initial phase (0–16 h) was also the major phase of l-lysine production (42.8% increase), which appeared growth coupled. TCA cycle-related intermediates (citrate, α-ketoglutarate) were utilized as major substrates during this initial phase. Carbohydrates were also taken up but at only a rather low rate. Dissolved oxygen was quickly consumed and became limiting after approximately 10 h, while the pH value significantly dropped. Acetate was the only by-product formed in significant amounts. Then, growth and l-lysine accumulation slowed (16–30 h). Likely linked to limited oxygen availability, fermentation products such as lactate and 2,3-butanediol emerged. With depletion of citrate and α-ketoglutarate, NCC 156 switched to consume mainly sucrose, while raffinose and stachyose degradation continued. After approximately 30 h, sucrose was used up, triggering an accelerated use of stachyose and raffinose (30–48 h). The level of maltose, on the other hand, increased. Overall, *B. amyloliquefaciens* NCC 156 showed an outstanding capability to degrade indigestible carbohydrates. Stachyose (94.8%) and raffinose (88.6%) were almost completely consumed.

**Fig. 5:**
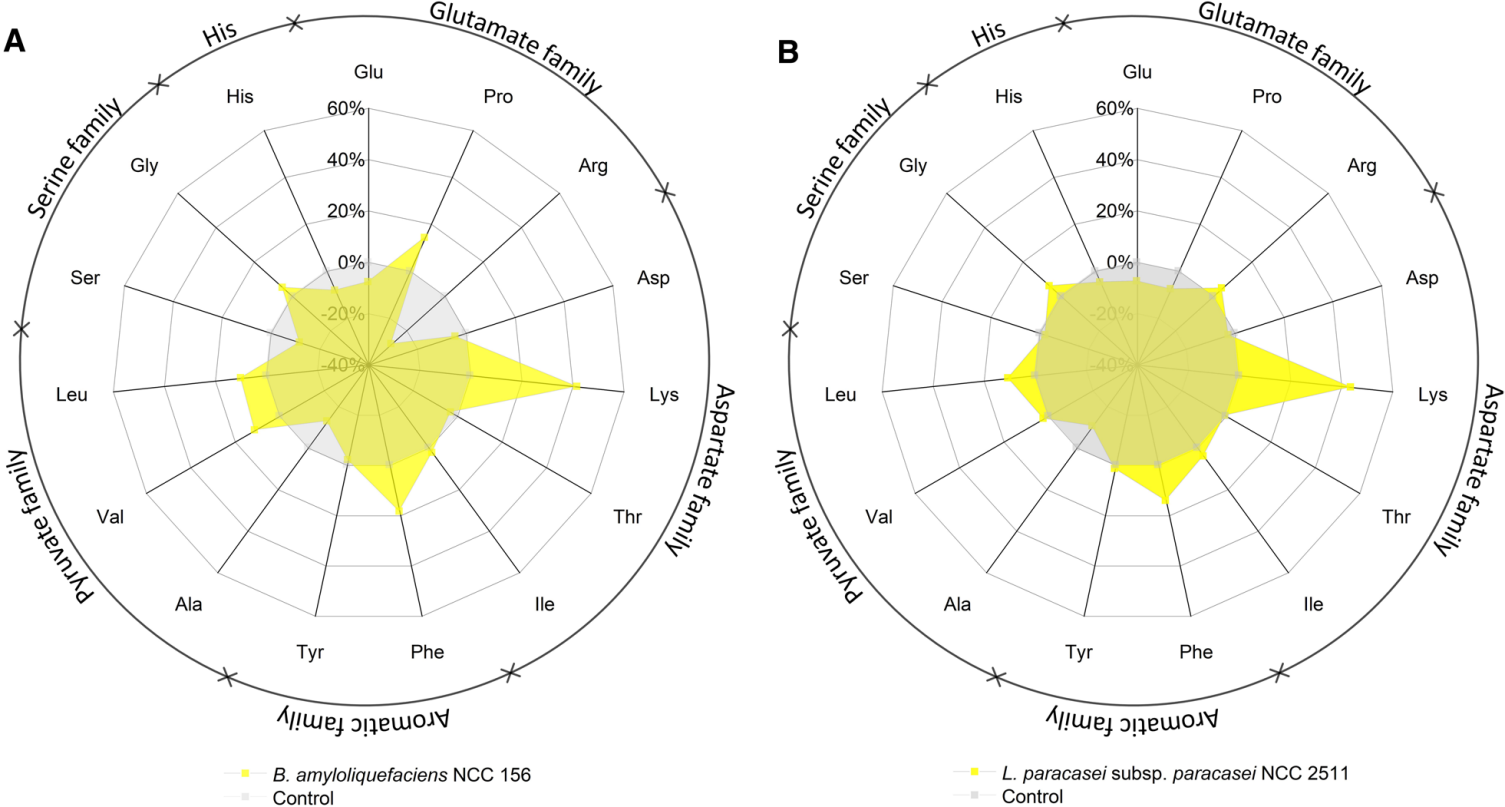
Relative changes in the amino acid profile during fermentation of chickpea milk using *B. amyloliquefaciens* NCC 156 (**A**) and *L. paracasei* subsp*. paracasei* NCC 2511 (**B**). n = 3

Remarkably, the formed l-lysine was largely present as a free amino acid. The extracellular l-lysine level increased almost ninefold to a final value of 1.4 mM after 24 h of fermentation. The pH value increased towards the end and finally again reached the starting value of approximately 6. At the end of the fermentation, acetate (40 mM), acetoin (30 mM), 2,3-butanediol (19 mM), and maltose (11 mM) were the main products. Ethanol, isobutyrate, and isovalerate, often fermentation products for other strains of *Bacillus*, were not detected. Regarding essential amino acids, the microbe increased the total levels of l-phenylalanine (18.6%), l-valine (11.1%), and l-leucine (10.0%) in addition to l-lysine, while the nonessential amino acids l-glutamate/l-glutamine, l-alanine, l-serine, and l-arginine – all fuelling the EMP pathway and the TCA cycle, respectively—were degraded (Fig. 5).

### Fermentation of chickpea milk using* L. paracasei* subsp*. paracasei* NCC 2511

*L. paracasei* subsp*. paracasei* NCC 2511 showed a completely different fermentation behaviour than *B. amyloliquefaciens* NCC 156 (Fig. 4). Its growth was much weaker, and changes in pH and dissolved oxygen were also less pronounced. Citrate was the preferred carbon source. It was completely depleted during the first hours, together with some of the α-ketoglutarate (18.2%). Sucrose (38.3%) and stachyose (16.2%) were partially consumed, while the raffinose level did not change, indicating a weaker ability of *L. paracasei* subsp*. paracasei* NCC 2511 to ferment carbohydrates. Acetate (10 mM) and lactate (2 mM) accumulated as by-products during the initial phase. Considering the generally lower metabolic activity, the high increase in total l-lysine (45.7%) during the first 24 h was remarkable for this strain. Interestingly, the formed l-lysine remained inside the cells and/or protein bound because the level of the free amino acid did not change significantly (data not shown). *L. paracasei* subsp*. paracasei* NCC 2511 slightly changed the amino acid profile (Fig. 5). The levels of l-phenylalanine (14.0%) and l-leucine (11.0%) increased, while l-glutamate/l-glutamine and l-alanine were partially consumed.

### Impact of fermentation on flavour development

**Fig. 6:**
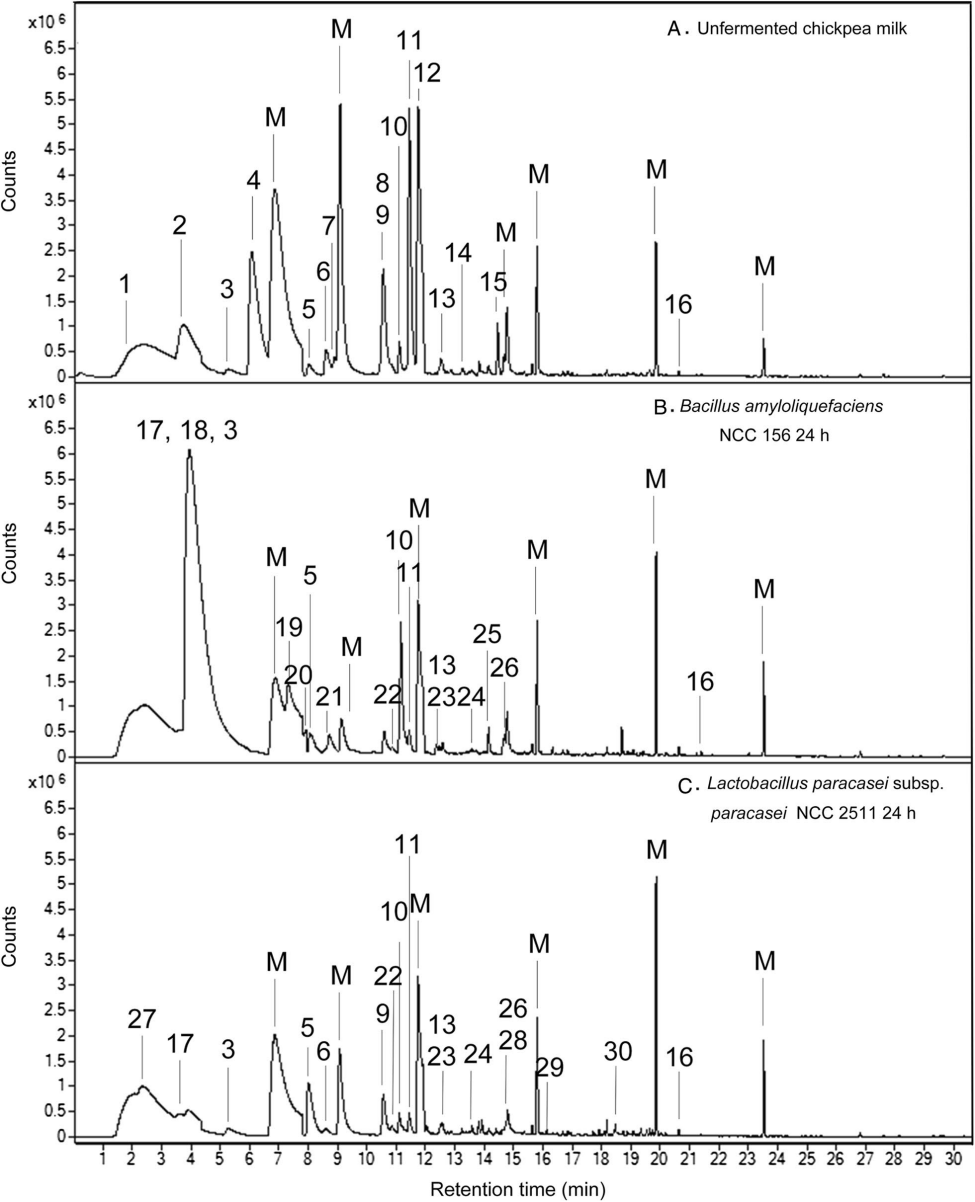
GC–MS-based analysis of volatile compounds in unfermented chickpea milk (**A**), chickpea milk fermented for 24 h with *B. amyloliquefaciens* NCC 156 (**B**) and chickpea milk fermented for 24 h with *L. paracasei* subsp*. paracasei* NCC 2511 (**C**). The peak numbers refer to the identified analytes: 1, acetone; 2, pentanal; 3, 1-pentanol; 4, hexanal; 5, 1-hexanol; 6, 2-heptanone; 7, heptanal; 8, cyclooctane; 9, benzaldehyde; 10, 1-octen-3-ol; 11, 2-pentylfuran, 12, octanal; 13, phenyl-methanol; 14, *trans*-2-octenal; 15, 1-nonanal; 16, γ-heptyl-butyrolactone; 17, acetoin; 18, isobutyric acid; 19, 2-methyl-hexanoic acid; 20, 2-methyl-butanoic acid; 21, acetoin acetate; 22, 1-heptanol; 23, ethyl-1-hexanol; 24, 1-octanol; 25, 2-nonanone; 26, maltol; 27, 2,3-butanedione; 28, 2-phenylethanol; 29, 1-nonanol; 30, nonanoic acid; M, peaks from sample matrix and instrumental background noise

**Fig. 7:**
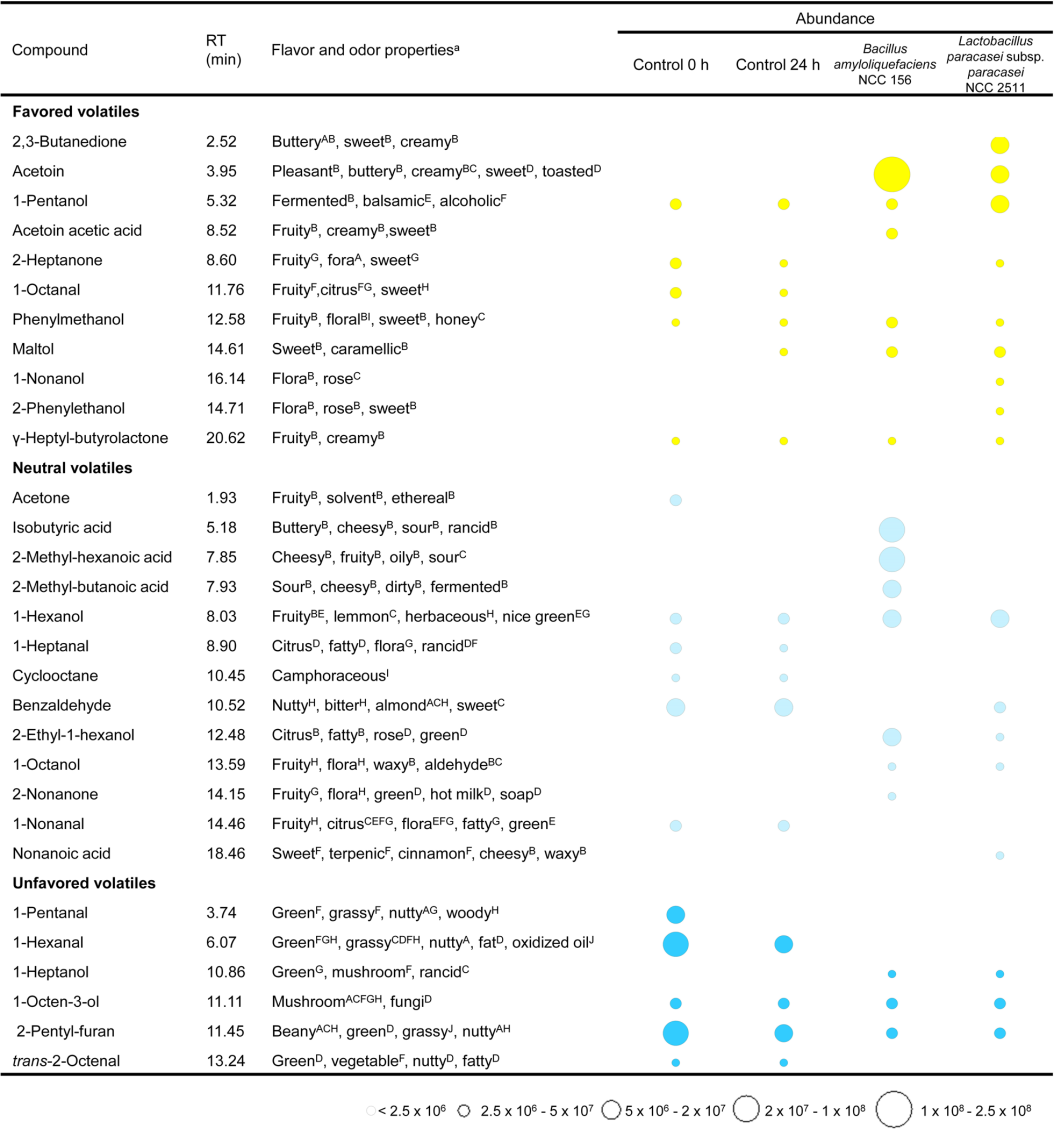
Aroma development during fermentation of chickpea milk with *L. paracasei* subsp*. paracasei* NCC 2511 and *B. amyloliquefaciens* NCC 156. The data reflect changes in the abundance of volatiles during 24 h of fermentation in comparison to their abundance in non-fermented chickpea milk, incubated under the same conditions (control). Volatile compound analysis was conducted using GC/MS, and compounds were identified based on their mass spectra using the NIST library. The given abundance reflects the mean peak areas from triplicate experiments. Compound classification into favoured volatiles with flora, fruity, sweet, and creamy aroma properties (yellow), neutral volatiles with concentration-dependent desired and undesired aroma properties (light blue), and unfavoured volatiles potentially contributing to the beany, green, and mushroom flavour (dark blue) relates to previous studies and databases on flavour [69, 99–101]. *RT* retention time. The assigned flavour properties are taken from previous studies and databases: **A** [102], **B** [103], **C** [76], **D** [104], **E** [105], **F** [69], **G** [106], **H** [107], **I** [108], **J** [109]. n = 3

GC–MS-based analysis revealed a strong impact of fermentation on the abundance of flavour-related volatiles (Fig. 6). In total, 30 volatiles were identified in unfermented and fermented chickpea milk, including various saturated and unsaturated organic alcohols, aldehydes, ketones, organic acids, lactones, and furans. In unfermented chickpea milk, volatiles with grassy and beany flavours, such as hexanal, pentanal, 1-octen-3-ol, *trans*-2-octenal, and 2-pentyl-furan, dominated, while sweet and fruity aroma compounds (e.g., 2-heptanone, octanal, and γ-heptyl-butyrolactone) were present in only low amounts. Fermentation with *B. amyloliquefaciens* and *L. paracasei* subsp*. paracasei* changed the flavour profile significantly, whereby each microbe yielded a unique signature at the end of the fermentation (Fig. 7). *L. paracasei* subsp*. paracasei* NCC 2511 produced various alcohols, such as 2-ethyl-1-hexanol, 1-pentanol, 1-hexanol, 1-heptanol, 1-octanol, 1-nonanol, and 2-phenyethanol. *B. amyloliquefaciens* generated mainly organic acids, including isobutyric acid, 2-methyl-butanoic acid, and 2-methyl-hexanoic acid. Aldehydes such as pentanal, hexanal, heptanal, and *trans*-2-octanal were eliminated by both strains, whereas 2-pentyl-furan was at least partially removed. *B. amyloliquefaciens* further consumed 2-heptanone and benzaldehyde. The unfermented control was largely unchanged, except for a certain degree of evaporation of some of the more volatile compounds, underlining the contribution of the microbes to the change in flavour compounds.

### Metabolic pathway analysis: l-Lysine biosynthesis

**Fig. 8:**
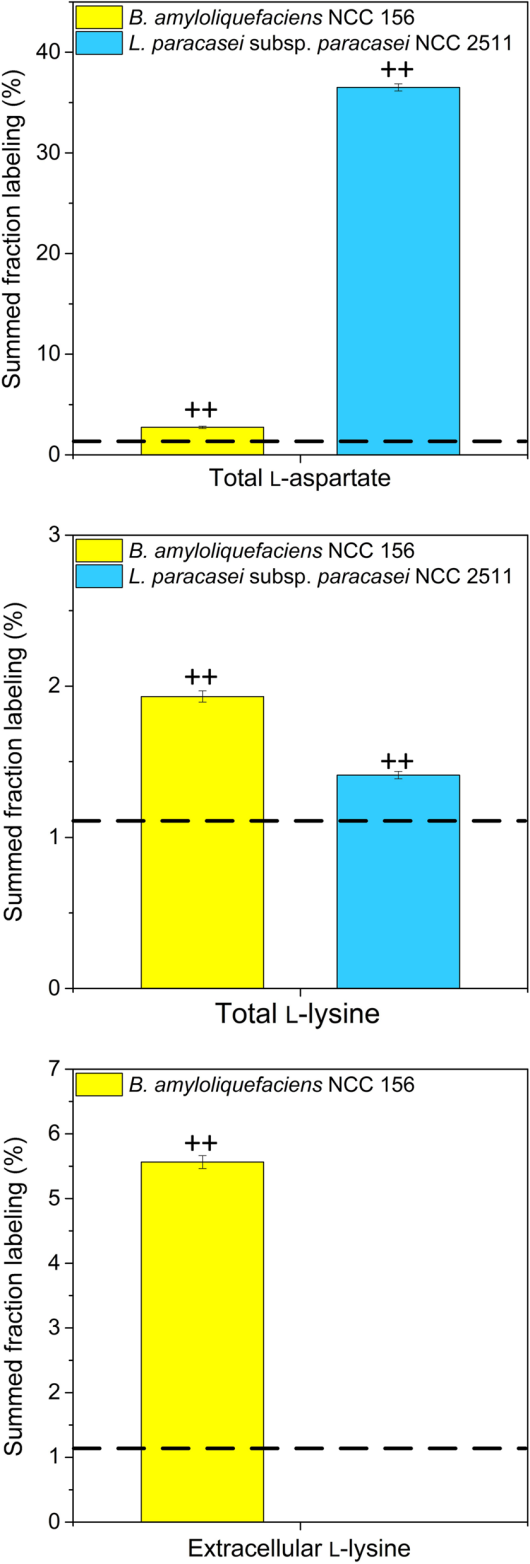
Metabolic flux analysis of l-lysine biosynthesis in *B. amyloliquefaciens* NCC 156 (yellow) and *L. paracasei* subsp*. paracasei* NCC 2511 (blue) during chickpea milk fermentation. The carbon flux through of l-lysine biosynthesis was investigated by monitoring the incorporation of ^13^C from [^13^C_4_] l-aspartate, added as a tracer to the medium, into l-lysine. As a control, naturally labelled l-aspartate was supplemented to the same amount instead. The data show the ^13^C enrichment in total l-lysine, free extracellular l-lysine, and total l-aspartate after 20 h of fermentation, measured by GC/MS. The given summed fractional labelling (SFL) reflects the average enrichment of ^13^C in each of the analytes. The corresponding values from the control experiment for total l-aspartate (1.1 ± 0.1%), total l-lysine (1.1 ± 0.1%), and free extracellular l-lysine (1.1 ± 0.1%) matched the theoretical values expected from natural ^13^C abundance. They are shown as dashed lines. The significance of ^13^C enrichment in each analyte was determined by comparison of the measured value against the naturally labelled control, and is marked accordingly (*p < 0.05, **p < 0.01). n = 3

It appeared interesting to study the l-lysine pathway in more detail. As inferred from the genomic repertoire, it contained l-aspartate as central intermediate (Fig. 1). Monitoring of the incorporation of ^13^C from [^13^C_4_] l-aspartate, added as a tracer substrate to the fermentation process, into l-lysine was used to elucidate biosynthesis in vivo. For this purpose, 11 mM [^13^C_4_] l-aspartate was added to medium. After 20 h of fermentation, the ^13^C enrichment of total l-lysine was analysed by GC/MS. In a control study, the medium was supplemented with the same amount of naturally labelled l-aspartate, and the ^13^C enrichment of total l-lysine was analysed. The ^13^C enrichment of total l-lysine in the control was 1.1 ± 0.1%, reflecting the expected natural ^13^C abundance [32]. Notably, it was significantly increased to a value of 1.9 ± 0.0% in the tracer study (p < 0.01) which indicated that [^13^C_4_] l-aspartate had been converted into l-lysine (Fig. 8). The ^13^C enrichment of extracellular l-lysine was even more increased (5.6 ± 0.1%), underlining that cells excreted large amounts of the free amino acid, formed from l-aspartate, into the media (Fig. 8). Parallel analysis of the ^13^C enrichment of l-aspartate revealed a substantial decrease from a high initial value of 38.5 ± 1.2% (0 h) to 2.7 ± 0.1% (20 h) indicating that the tracer was largely used up during this time (Fig. 8). In a parallel study, de novo biosynthesis of l-lysine was also observed for *L. paracasei subsp. paracasei* NCC 2511. Using [^13^C_4_] l-aspartate as tracer, the ^13^C enrichment of total l-lysine after 20 h was again significantly increased (1.4 ± 0.0%, p < 0.01) (Fig. 8). On a first glance, it appeared less pronounced. The weaker increase in ^13^C enrichment in l-lysine than in NCC 156, however, partially resulted from the fact that the added [^13^C_4_] l-aspartate was only slightly used, i. e. the ^13^C enrichment of total l-aspartate after 20 h (36.5 ± 0.4%) was almost as high as the starting value (38.5 ± 1.2%) indicating only little consumption (Fig. 8).

As shown above, citrate appeared to be the preferred carbon source of both strains, and, for *B. amyloliquefaciens*, its consumption happened simultaneously with l-lysine formation, suggesting a potential link (Fig. 4). In additional studies, citrate (10 mM) was spiked into the medium. For *B. amyloliquefaciens* NCC 156, this resulted in 27% increased l-lysine production to 1.8 ± 0.2 mM after 24 h, while the growth of the microbe was stimulated to 10^9.5^ cfu mL^−1^. For *L. paracasei* subsp*. paracasei*, no significant impact on growth or l-lysine formation was observed (data not shown).

**Fig. 9:**
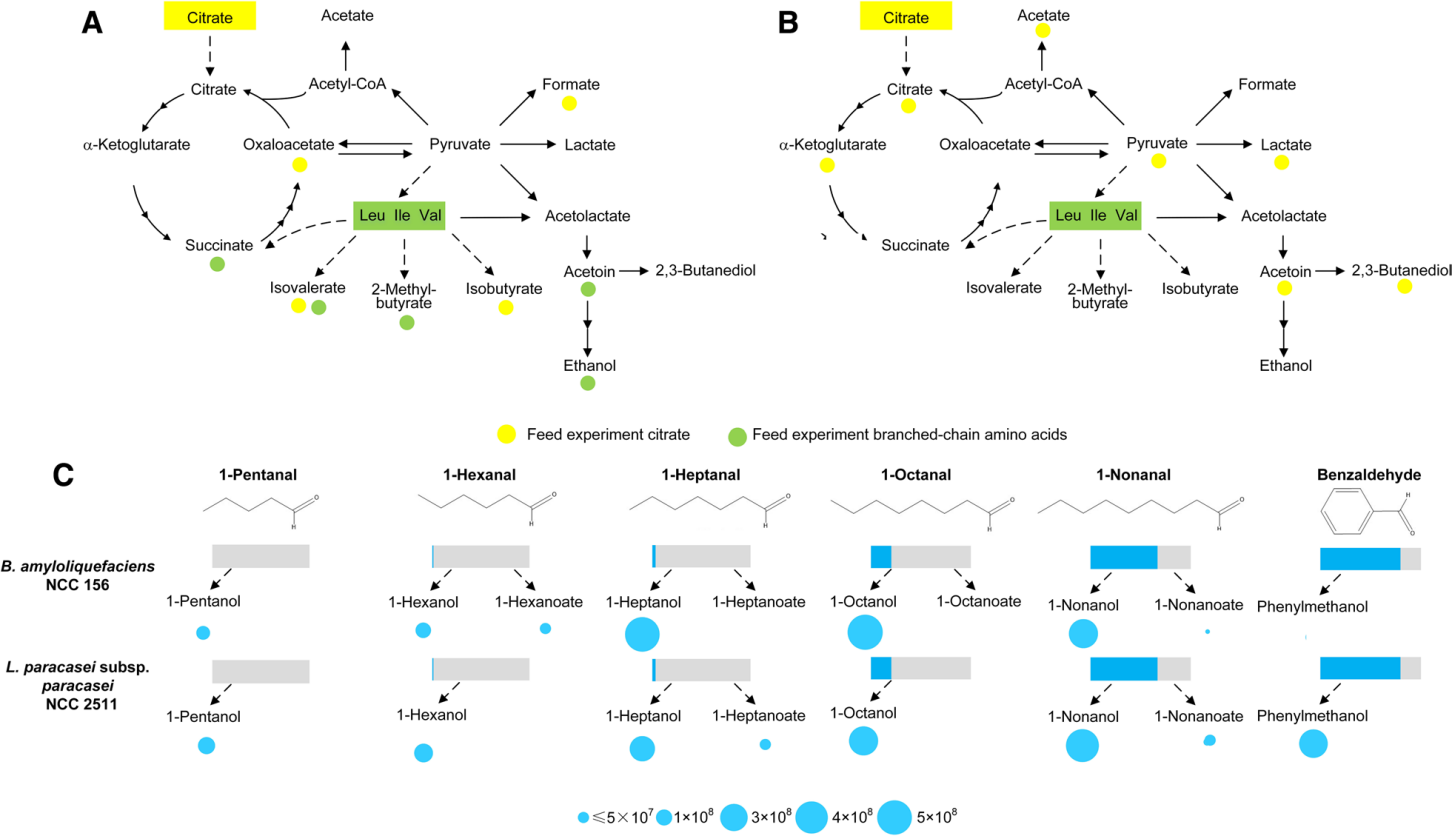
Metabolic pathway analysis in *B. amyloliquefaciens* NCC 156 and *L. paracasei* subsp*. paracasei* NCC 2511. Metabolism of citrate and branched-chain amino acids (**A**, **B**) and of short- to medium-chain flavour aldehydes (**C**, **D**). The data reflect changes in metabolite abundances during 24 h of fermentation in comparison to those in non-fermented chickpea milk incubated under the same conditions (control). In separate experiments, citrate (yellow, 10 mM), a mixture of the branched-chain amino acids valine, leucine, and isoleucine (green, 5 mM each), and flavour aldehydes (blue, 5 mM, each tested individually in separate experiments) were spiked into chickpea milk. The evaporation of aldehydes (assessed from non-inoculated controls) is indicated as relative loss by the grey colour. The formation of alcohols and acids from a particular aldehyde is visualized by the size of the corresponding circle associated with the products (p < 0.05). n = 3

The genome of *L. paracasei* subsp*. paracasei* contained a potential citrate degradation route via citrate lyase, while that of *B. amyloliquefaciens* NCC 156 did not (Additional file 1: Table S4). The latter observation suggested that, in this microbe, citrate was apparently converted via the TCA cycle towards oxaloacetate and the precursor of l-lysine instead (Fig. 9).

### Metabolic pathway analysis: Fermentation by-products and flavour molecules

The citrate supplemented cultures (see above) were now evaluated for citrate-related effects on formation of flavour molecules and fermentative by-products. Both microbes differed in the genomic repertoire regarding these parts of metabolism (Additional file 1: Table S4). Citrate contributed to the formation of different organic acids in *L. paracasei* subsp*. paracasei*, including acetate, lactate, pyruvate, and α-ketoglutarate, when spiked into chickpea milk (Fig. 9AB). In addition, acetoin and 2,3-butanediol were obviously produced from citrate. *B. amyloliquefaciens* formed higher amounts of short-chain fatty acids (such as isovalerate and isobutyrate) from citrate.

In a series of further experiments, different chickpea milk contained aldehydes (Figs. 6, 7)—flavour compounds themselves and precursors for other flavours as well—were individually spiked into the cultures. Both microbes efficiently reduced pentanal, hexanal, heptanal, octanal, nonanal, and benzaldehyde into the corresponding alcohols (Fig. 9C). *L. paracasei* subsp*. paracasei* NCC 2511 further revealed a significant capability to oxidize five-carbon to nine-carbon aldehydes into the corresponding acids. *B. amyloliquefaciens* NCC 156 also oxidized aldehydes, more selectively the odd chain compounds pentanal, heptanal and nonanal. Moreover, we tested flavour-related effects by supplementing branched chain amino acids (Fig. 9AB). *B. amyloliquefaciens* NCC 156 degraded leucine, isoleucine, and valine (spiked into the medium) into short chain fatty acids, whereas *L. paracasei* subsp*. paracasei* did not metabolize these amino acids.

## Discussion

### Genome-based selection appears to be an efficient strategy to identify l-lysine overproducers for chickpea milk fermentation

Since the early days of humankind, fermentation has been a natural approach to produce food, and today, fermented foods are more popular than ever [33, 34]. Given the magnitude of available microbes for plant milk fermentation (or even food fermentation in general), straightforward selection of the most appropriate microbes appears crucial. Previous approaches that successfully increased the l-lysine content in chickpea [16], soybean [13–15, 35], mung bean [17], and cowpea [18] found the corresponding microbes mainly only spontaneously and occasionally.

Here, potential l-lysine producers were systematically selected, based on their genomic repertoires. By strict filtering of genome sequences for key genes related to l-lysine synthesis and degradation, an initially high number of approximately 2,500 potential candidates was narrowed down to 31 strains that possessed the key genes *lys A* and *dapF* for l-lysine biosynthesis, incomplete pathway sets for l-lysine degradation and fulfilled the QPS recommendation. The selected strains represented 10 different microbial genera and (given the low number) enabled straightforward experimental work. As a valuable proof-of-concept, 30% of all experimentally tested strains exhibited the desired phenotype in increasing the l-lysine content, and it appears promising to extend this genome-based selection approach to other plant milk fermentation processes and traits. Although our study could not yield a complete picture, it provided at least systematic insight into the pathways that supported l-lysine production most. Among the strains that exhibited growth and l-lysine accumulation, the majority (79%) possessed a completely annotated acetylase pathway (AP), while 32% exhibited a complete succinylase pathway (SP), and two isolates had both routes in parallel. None of the strains used here contained the dehydrogenase pathway (DP). The different biosynthetic routes are widely abundant in nature: there are SP-using species such as *Escherichia coli*, *Corynebacterium*, *Bacillus,* and *Lactobacillus* [36–38], AP users such as certain *Bacillus* and *Lacticaseibacillus* [38, 39], and DP users such as *Corynebacterium* and some *Bacillus* species [36]. The AP and SP both supported l-lysine accumulation in chickpea milk, with a slightly better performance from the AP.

Among catabolic pathways and potential l-lysine withdrawal and degradation, there was no clear trend. The competing routes to homoserine [40, 41] and peptidoglycan [42, 43] did not have an impact. However, *B. pumilus* NCC 2962, the only strain tested that possessed a lysine decarboxylase (EC. 4.1.1.18)-encoding gene (Additional file 1: Fig. S2), dramatically decreased the l-lysine content so that future strain selection might consider potentially negative effects of this enzyme. Taken together, AP and SP genes appear to be genomic key features of well-performing l-lysine-producing strains. Based on performance, future focus should be given to strains of *Lacticaseibacillus, Limosilactobacillus, Levilactobacillus,* and *Bacillus.* Given that these genera are among the most widely used microbes for plant-based fermentation [44, 45], there seems striking potential ahead.

### *B. amyloliquefaciens* NCC 156 and *L. paracasei* subsp*. paracasei* NCC 2511 are drivers of a multi-benefit chickpea milk fermentation

Plant-based milk alternatives preferably exhibit the technical, nutritional, and organoleptic properties of cow milk, so researchers and developers in academia and industry must overcome certain challenges [1]. From a technical viewpoint, gelation of typically starchy plant materials during sterilization causes problems in downstream processing [46]. Conventional autoclaving and pasteurization failed as pre-treatment strategies due to the reasons mentioned above. The developed two-step heat treatment, however, provided a homogeneous and stable emulsion and was a valuable strategy for processing chickpea milk prior to fermentation at a small scale.

On the fermentation side, several goals had to be addressed: (i) improvement of the naturally low nutritional value of plant milks limited in amino acids [47], (ii) improvement of digestibility to avoid flatulence, diarrhoea, and other discomforts [48], and (iii) improvement of the typically unpleasant earthy and beany taste [49]. It is therefore an important outcome of this study that *B. amyloliquefaciens* NCC 156 and *L. paracasei* subsp*. paracasei* NCC 2511 addressed several of these major criteria well. As shown, they increased the l-lysine level by up to 43% (Fig. 3), largely removed indigestible sugars (Fig. 4EL), improved the amino acid profile (Fig. 4), and improved the flavour (Fig. 7). Based on their performance, they are well-performing microbes for chickpea milk fermentation. The multiple benefits delivered, and the food-grade approval associated with both strains suggest their great potential for use in industry.

In this regard, *B. amyloliquefaciens* NCC 156 and *L. paracasei* subsp*. paracasei* NCC 2511 stand in a prominent line with related microbes. For example, *B. amyloliquefaciens* strains are found in fermented legume-based foods, including doenjang and meju [50, 51], and are often regarded as probiotics due to their beneficial effects on functional food products [52]. Selected bacilli improved the l-lysine content in soy-based food [14, 53, 54]. *L. paracasei* subsp*. paracasei* NCC 2511 belongs to the group of facultative heterofermentative lactic acid bacteria (LAB), which are also widely used in dairy and plant-based milk fermentation [55–57] and known to exhibit probiotic properties [58, 59] and increase l-lysine levels, as shown for soy meal [60], soybean flour [14], and cowpea milk [18].

The efficient consumption of (also indigestible) oligosaccharides and the accumulation of maltose by *B. amyloliquefaciens* NCC 156 (obviously resulting from degraded starch) revealed its high glycosidic activity involving α-galactosidase, β-fructosidase, and a-amylase (Fig. 4DE, Additional file 1: Table S4) The enzymatic portfolio, also observed in other bacilli [61, 62], appeared valuable since it improved the digestibility and acceptability of the fermented product. *L. paracasei* subsp*. paracasei* NCC 2511 also exhibited β-fructosidase, α-galactosidase, and α-amylase activity but to a weaker extent (Fig. 4KL, Additional file 1: Table S4). According to previous studies, the glycolytic capacity among strains of *L. paracasei* subsp*. paracasei* and other lactobacilli is rather strain specific [4, 57, 63–66].

Interestingly, the two microbes utilized nutrients differently. As a striking example, citrate stimulated the growth and l-lysine production of *B. amyloliquefaciens* NCC 156, which is an interesting finding given that sugars are mainly known to drive l-lysine overproduction [41]. In contrast, citrate triggered aroma development in *L. paracasei* subsp*. paracasei* NCC 2511. Hereby, the organic acid was obviously converted into four-carbon dicarboxylic acids such as succinate and oxaloacetate via the TCA cycle and potentially also via citrate lyase and then triggered the production of acetate, acetoin, acetate, and smaller amounts of lactate [67, 68]. *B. amyloliquefaciens*, lacking a citrate lyase pathway, instead used acid for growth.

### A rich set of microbial aroma compound conversions contributes to an improved flavour profile during chickpea milk fermentation

Flavours are a major attribute that consumers consider when buying plant milk-derived products [69]. Unfortunately, plant-based milk alternatives are generally perceived as having a displeasing taste, probably because of previous experiences with less-appealing products in the market [49]. As shown here, unfermented chickpea milk contained various volatiles associated with an undesired taste (Fig. 7), a typical drawback for these types of plant-based raw materials [70]. Prominent beany and earthy off-flavours are caused by medium-chain aldehydes such as pentanal, hexanal, and heptanal [71], which originate from the oxidation of plant lipids and are also found in high amounts in other legume-based milks [71].

It can therefore be regarded as beneficial that *L. paracasei* subsp*. paracasei* NCC 2511 and *B. amyloliquefaciens* NCC 156 largely eliminated these aldehydes (Fig. 9C). As shown by spiking experiments, the major routes of elimination seemed to be one-step reductive and oxidative biotransformations into the corresponding alcohols and acids, respectively, mostly yielding a strong upgrade in flavour properties into sweet and fruity notes [72], although multistep bioconversion and de novo formation found in other microbes appeared possible as well [73].

Other flavour-related conversions involved entire pathways of carbon core metabolism, underlining the complexity involved. Citrate and branched-chain amino acids were identified as important precursors. Citrate metabolism in *L. paracasei* subsp*. paracasei* provided acetate, lactate, acetoin, and 2,3-butanediol, whereas valine, leucine and isoleucine degradation delivered isobutyrate, isovalerate, and 2-methylbutanoate. Based on the discovered link between the chickpea ingredient and specific flavours, screening for plant-based materials rich in such precursors to stimulate the formation of desired flavour notes appears promising [74]. As demonstrated, elevated citrate levels would also provide more l-lysine. An interesting possibility also seems to be the direct addition of such compounds to fermentation to generate beneficial fermentation products.

Maltol is also formed, and it contributes to a sweet caramel flavour and is released by the cleavage of antinutrient saponins during fermentation [75]. The metabolic origin of the removal of other compounds, such as 1-octen-3-ol and 2-pentyl-furan, remains to be elucidated, but their degradation surely reduces the undesired earthy and beany taste of chickpea flour [76]. Clearly, the overall aroma of (fermented) legume-based milk is not due to single chemicals but rather their exact mixture [70]. As such, the overall decrease in off-odour aldehydes plus the formation of favoured sweet and fruity alcohols, acids, ketones, and phenols is an important indication that sensory properties were significantly improved, but more work, e.g., using sophisticated electronic aroma sensing and personal aroma detection by skilled and trained sensory panellists, is needed to fully capture the underlying changes [71, 77, 78].

## Conclusions

In this work, a genome-based approach supported the selection of two well-performing strains for the fermentation of chickpea milk. As shown, each of the microbes exhibited multiple benefits: an improved l-lysine content due to de novo synthesis of the amino acid, which was proven in ^13^C experiments; an improved digestibility due to the removal of raffinose and stachyose; and an improved flavour profile due the elimination of off-flavour aldehydes and the generation of sweet and fruity aromas. The origins of most of the desired traits were found at the metabolic pathway level to prove the microbial contributions, which we regard as important and valuable in terms of understanding the observed benefits as the results of natural processes catalysed by safe microbes, suggesting greater use of systems biology approaches [32, 79–81] to study food fermentation. In particular, the use of ^13^C isotopes for flux analysis, applicable on a small scale [82], appears valuable in this regard.

Notably, each of the two microbes exhibited a unique signature. *B. amyloliquefaciens* NCC 156 showed a greater capability to grow, likely enabled by its stronger portfolio of hydrolytic enzymes, whereas *L. paracasei* subsp*. paracasei* NCC 2511 exhibited pronounced synthesis of selected beneficial molecules. In this regard, each of the two strains has its own benefits for use in fermentation of chickpea milk and appears promising for testing on other plant-based milks.

## Material and methods

### Microorganisms

The microbial strains used in this work were obtained from the NCC (Nestlé Research Centre, Lausanne, Switzerland) (Additional file 1: Table S1). All strains were food-grade approved based on the qualified presumption of safety (QPS) recommendation [22]. They were maintained as frozen stocks in 30% glycerol (v/v) at − 80 °C.

### Genome-based strain selection

The gene sequence information for strain selection was obtained from the NCC genome database [83]. The database was screened for the presence and absence of genes of interest using automated annotation. A proprietary database of enzyme domain profiles was used to screen all NCC genome proteins using NCBI's Reverse Position Specific Basic Local Alignment Search Tool (RPS-BLAST) algorithm, constrained by minimum query coverage (60%), minimum hit coverage (60%, [84]. A particular enzyme domain was regarded as present when the corresponding E-value was below 1E-3.

### Pre-culture medium

Depending on individual nutrient requirements, different media were used for pre-cultivation of the strains. Most lactobacilli (*L. helveticus*, *L. hilgardii, L. delbrueckii, L. plantarum, L. brevis, L. johnsonii, L. reuteri, L. paracasei,* and *L. acidophilus*) were cultivated in de Man‐Rogosa‐Sharpe (MRS) medium, containing 52.0 g of MRS broth (Sigma-Aldrich, Steinheim, Germany) and 1.0 mL of Tween-80 per litre [85]. *F. sanfranciscensis* and *L. pontis* were grown in sanfrancisco medium [86], which was modified in this work and contained 10.0 g of tryptone (Becton Dickinson, Franklin Lakes, NJ, USA), 7.0 g of yeast extract (Becton Dickinson), 7.0 g of glucose, 7.0 g of fructose, 7.0 g of maltose monohydrate, 5.0 g of sodium acetate trihydrate, 5.0 g of di-ammonium hydrogen citrate, 2.5 g of KH_2_PO_4_, 2.0 g of Lab Lemco powder (Oxoid, Thermo Fisher, Rockford, IL, USA), 2.0 g of sodium gluconate, 0.5 g of l-cysteine hydrochloride, 0.4 g of MgSO_4_·7H_2_O, 0.1 g of MnSO_4_·H_2_O, 0.05 g of FeSO_4_·7H_2_O, and 1.0 mL of Tween-80 per litre. All *Bifidobacterium* strains were grown in MRS-Cys broth containing 52.0 g of MRS broth, 0.5 g of cysteine hydrochloride, and 1.0 mL of Tween-80 per litre. The strains of *Bacillus* were grown in modified tryptic soy broth containing 17.0 g of tryptone (Becton Dickinson), 5.0 g of NaCl, 3.0 g of soytone (Becton Dickinson), 2.5 g of K_2_HPO_4_, and 1.0 mL of 30% silicone antifoam (Sigma-Aldrich) per litre. *C. stationis* was cultivated in brain heart infusion cysteine (BHI-Cys) broth containing 37.0 g of brain heart infusion (Becton Dickinson) and 0.5 g of l-cysteine hydrochloride per litre.

### Chickpea milk medium

A chickpea suspension was prepared by mixing 10% (w/w) commercially produced chickpea flour (E. Zwicky AG, Müllheim-Wigoltingen, Switzerland) with deionized water. Flour from one batch (25 kg) was used in all experiments. A two-step heat treatment was applied for sterilization. First, the suspension was stirred (250 rpm, 2 h, 75 °C), which was followed by autoclaving (121 °C, 15 min). Prior to fermentation, the suspension was manually homogenized. In ^13^C tracer experiments, 98% [^13^C_4_] l-aspartate (Eurisotop, Gif-Sur-Yvette, France) was added to a final concentration of 11 mM, whereas controls received the same amount of naturally labelled l-aspartate. In further experiments, selected nutrients were added to chickpea milk from sterilized stocks before fermentation to investigate their impact: (i) citrate (10 mM), (ii) a mixture of the branched-chain amino acids valine, leucine, and isoleucine (5 mM each), and (iii) volatile aldehydes such as pentanal, hexanal, heptanal, octanal, nonanal, and benzaldehyde (5 mM each in separate experiments).

### Chickpea milk fermentation

Strains were inoculated from a glycerol stock (200 µL) into 10 mL of pre-culture medium and incubated overnight. The medium and growth temperatures were individually adapted (Additional file 1: Table S1). In addition, different conditions were used to grow obligate anaerobic, aerotolerant and obligate aerobic strains (Additional file 1: Table S1). Obligate anaerobic strains were incubated in 20 mL tubes filled with 10 mL of pre-culture medium and placed in an anaerobic jar at 9—13% CO_2_ (anaerobic atmosphere generation bags, Merck, Darmstadt, Germany). Aerotolerant strains were cultivated in the same tubes (20 mL filled with 10 mL of medium) but placed under ambient air. Obligate aerobic strains were cultivated in 10 mL of pre-culture medium in 100 mL non-baffled shake flasks on a rotary shaker (130 rpm, 80% humidity, Infors, Bottmingen, Switzerland). Subsequently, cells were collected (5,000 × *g*, 5 min, 4 °C) and used to inoculate a second pre-culture grown under the same conditions overnight, which was then used as inoculum for the main chickpea milk fermentation. Depending on the studied microbe, the main fermentation was then conducted in 20 mL of medium in 200 mL glass bottles under a CO_2_ atmosphere (obligate anaerobes) or ambient air (aerotolerant) and in 100 mL non-baffled shake flasks (obligate aerobes).

Potential evaporation loss during fermentation was monitored using non-inoculated controls and used to correct the obtained data [32]. The non-inoculated cultures further served as controls to verify the sterility of the raw material and monitor the occurrence of abiotic changes that potentially altered the composition. In selected experiments, the pH value and dissolved oxygen (DO) level were monitored in shake flasks with immobilized sensor spots and fluorescence detection (PreSens Precision Sensing GmbH, Regensburg, Germany) [87]. Each fermentation was conducted as biological triplicates.

### Quantification of colony-forming units

The estimation of cfu was based on plate serial dilution spotting [88]. Serial dilutions from a 1 mL sample were prepared in 0.85% NaCl (w/v) containing 1 g L^−1^ tryptone (Becton Dickinson). All measurements were conducted in duplicate.

### Quantification of chickpea milk moisture

Samples of 2 g were freeze-dried for 48 h. Then, the remaining total solids were quantified by measuring the residual weight.

### Protein and amino acid quantification

For the quantification of total amino acids, samples were homogenized (2 mL Precellys Lysing Kits filled with 1.4 mm ceramic balls, Bertin, Montigny-le-Bretonneux, France) using a homogenizer (5000 rpm, 3 × 90 s with 30 s breaks on ice in between). Approximately 10 mg of a homogenized sample was then hydrolysed (24 h, 6 M HCl, 100 °C), dried, resuspended in deionized water, and filtered (0.22 µm, Millipore, Merck, Darmstadt, Germany). The obtained amino acids were analysed by HPLC (Agilent 1100 Infinity, Agilent Technologies, Waldbronn, Germany) with pre-column derivatization with *ortho*-phthaldialdehyde and 2-aminobutyrate as an internal standard [89]. In short, the analytes were separated at 40 °C and a flow rate of 1 mL min^−1^ on a reversed-phase column (Gemini 5 µm C18 110 Ǻ, 150 × 4.6 mm, Phenomenex, Torrance, CA, USA) using the following gradient of eluent A (40 mM Na_2_HPO4, 7.7 mM sodium azide, pH 7.8) and eluent B (45% acetonitrile, 45% methanol, 10% deionized water): 0—45% B from 0 to 45 min, 45 to 61% B from 45 to 47 min, 61 to 82% B from 47 to 48 min, 82 to 100% B from 48 to 48.5 min, 100% B from 48.5 to 50.5%, and 100 to 0% B from 51 to 53 min. The analytes were detected by fluorescence (340/450 nm). l-cysteine, l-methionine, and l-tryptophan were degraded during the hydrolysis process so that these amino acids were not measurable, whereas l-glutamine and l-asparagine were converted into l-glutamate and l-aspartate, respectively, so that the obtained data reflected the lumped pools [90]. The total protein amount was calculated by summing up the level of the individual amino acids. For the quantification of free amino acids, 1 mL fermentation sample was centrifuged (20,000 × *g*, 10 min, 4 °C). The obtained supernatant was filtered (0.22 µm, Millipore) and analysed as described above.

### Quantification of sugars, organic acids, and alcohols

Disaccharides (sucrose and maltose) and larger oligomers (raffinose, stachyose, and verbascose) were quantified in filtered samples (0.22 µm, Millipore) using HPLC (Agilent 1260 Infinity Series, Agilent Technologies). The analytes were separated on a sulfonated spherical PS/DVB resin (VA 300/7.8 Nucleogel sugar Pb, Macherey–Nagel, Düren, Germany) with deionized water as the mobile phase (80 °C, 0.4 mL min^−1^) and detected via the refraction index. External standards were used for quantification. The concentrations of organic acids (lactate, acetate, pyruvate, citrate, α-ketoglutarate, isobutyrate, isovalerate) and alcohols (acetoin, ethanol, 2,3-butanediol) in filtered samples (0.22 µm, Millipore) were determined using HPLC (Agilent 1260 Infinity Series, Agilent Technologies) with ion exclusion (Aminex HPX-87H, 300 × 7.8 mm, Bio-Rad, Hercules, CA, USA), 12 mM H_2_SO_4_ as the mobile phase (45 °C, 0.5 mL min^−1^), and refractive index detection [91]. External standards were used for quantification.

### Quantification of lipids

Analysis of the lipid (fat) content was conducted after hexane/isopropanol extraction [92]. Briefly, 5 mL of hexane/isopropanol (3:2) was added to a 1 mL sample. The mixture was incubated for 24 h at 18 °C and 230 rpm. After addition of 5 mL of Na_2_SO_4_ (0.47 M), the extract was centrifuged at 4000 × *g* for 10 min. The upper liquid phase was collected and extracted once more using 5 mL of hexane/isopropanol (7:2). Both lipid fractions were then combined and dried under nitrogen flow until a constant weight was reached. The remaining solids were weighed and used to calculate the lipid content.

### Mass isotopomer analysis by GC–MS and processing of ^13^C data

In isotopic tracer experiments, the ^13^C labelling patterns of l-lysine and l-aspartate were analysed using GC–MS [32]. For ^13^C labelling analysis of the total amino acids, fermentation samples were homogenized as described above. Approximately 10 µL of a homogenized sample was hydrolysed (24 h, 6 M HCl, 100 °C), filtered (0.22 µm, Millipore), dried under a nitrogen flow, and dissolved in 50 μL of N,N-dimethylformamide (1% (v/v) pyridine). For ^13^C analysis of free l-lysine, a 1 mL sample was centrifuged (20,000 × *g*, 10 min, 4 °C). An aliquot of 50 µL of the obtained supernatant was filtered (0.22 µm, Millipore), dried under a nitrogen flow, and dissolved in 50 μL of N,N-dimethylformamide (1% (v/v) pyridine). The amino acids were then derivatized at 80 °C for 30 min into the corresponding *t*-butyldimethylsilyl derivatives using 50 μL of N-methyl-*t*-butyldimethylsilyl-trifluoroacetamide (MBDSTFA, Macherey–Nagel). Mass spectrometric analysis was conducted on a GC–MS instrument (Agilent 5977 A MSD, Agilent Technologies) equipped with an HP-5MS column (30 m, 0.250 mm, 0.25 µm, Agilent Technologies) using helium as the carrier gas (1.7 mL min^−1^) and the following temperature gradient: 120 °C (0–2 min), 8 °C min^−1^ (2–12 min), 10 °C min^−1^ (12–24.5 min), and 325 °C (24.5–27 min). The [M-57] ion cluster of the derivatized amino acids (*m/*z 431 for l-lysine, *m/z* 418 for l-aspartate) was selected to determine the ^13^C labelling pattern, as it represented the entire carbon backbone [90]. All samples were first measured in scan mode to exclude isobaric matrix interference with the analytes of interest and verify that the selected ion clusters were suited for ^13^C quantification. Subsequently, labelling patterns were determined in duplicate using selective ion monitoring. The derived mass isotopomer distributions were corrected for natural isotopes [93] and used to derive the SFL [32]. The SFL expressed the labelling pattern as the average ^13^C enrichment of the included carbon. For method validation, naturally labelled samples were treated and analysed as given above.

### GC–MS analysis of volatile flavour and fragrance compounds

Analysis of volatiles was based on headspace solid-phase microextraction GS-MS (HS–SPME–GC–MS) [94] using a PAL RSI 120 autosampler (CTC Analytics, Zwingen, Switzerland) coupled to a GC–MS instrument (Agilent 8890 GC system, Agilent Technologies). Samples were immediately processed after collection. Approximately 5 mL of broth was amended with 1 g of NaCl and incubated for 20 min at 40 °C in a 20 mL sample vial at a shaking rate of 400 rpm. Afterwards, an SPME fibre (coated with 65 µm divinylbenzene/polydimethylsiloxane and preconditioned for 1 h at 260 °C, Agilent Technologies) was exposed to the headspace of the vial at a depth of 40 mm for 20 min to absorb the volatiles. Then, the fibre was introduced and incubated in the injection port of the GC–MS instrument (300 °C, 3 min) for desorption. The injector was operated in splitless mode. The analytes were separated on an HP-5MS column (30 m, 0.250 mm, 0.25 µm, Agilent Technologies) using helium as the carrier gas (0.4 mL min^−1^) and the following temperature gradient: 35 °C for 3 min, increase to 210 °C at 6 °C min^−1^, and 210 °C for 10 min. Chromatograms were recorded by monitoring the total ion current (TIC) over a mass range of 30 to 300 m*/z*. Following signal deconvolution (Agilent Chemstation, Agilent Technologies), analytes were identified based on their mass spectra against the NIST/EPA/NIH Mass Spectral Library (NIST 08), and corresponding area counts were collected for quantification. Where needed, synthetic standards were used to support the identification. All analytics were conducted in triplicate.

### Data processing and statistical analysis

All results displayed in Figures and Tables are shown as the mean values ± standard deviations (SDs). Statistical evaluation of the data was conducted by one-way analysis of variance (ANOVA), followed by Fisher's least significant difference (LSD) test and Duncan’s test. Differences in values were considered significant when the P value was less than 0.05 ( +) and 0.01 (+ +). Statistical analyses were performed using SPSS (version 24.0).

## Supplementary Information

**Additional file 1**: **Table S1**. Growth conditions and media used to pre-culture the different strains. **Table S2**. Microbial growth during chickpea milk fermentation. The data are given as (colony forming units) mL-1. n=3. **Table S3**. The number of locus in B. amyloliquefaciens NCC 156 and L. paracasei subsp. paracasei NCC 2511 encoding functional enzymes invoving in flavor formation,pyruvate, and butanoate metabolism. **Table S4**. The number of locus in B. amyloliquefaciens NCC 156 and L. paracasei subsp. paracasei NCC 2511 encoding functional enzymes invoving in carbohydrate degradation and citrate metabolism. **Fig. S1**. Pre-treatment strategies for the processing of chickpea flour suspensions prior to microbial fermentation. Phase separation for the untreated milk (A), contamination of non-inoculated milk after pasteurization (63°C, 5 h) (B), gelation and phase separation after stronger heating (90°C, 30 min) (C), gelation and phase separation after autoclaving (121°C, 15 min), generation of homogeneous and sterile suspension after a two-step treatment that included combined heating and stirring (2 h, 75°C, 250 rpm) and autoclaving (121 °C, 15 min) (E). The sterility of pasteurized milk (A) and two-step treated milk (D), was evaluated by non-inoculated incubation for 48 h at 37°C. **Fig. S2**. Genomic repertoire of food-grade microbes linked to l-lysine metabolism: pathways for l-lysine biosynthesis (LSP), pathways competing with l-lysine biosynthesis for carbon precursors (PCP), and pathways for l-lysine degradation (LDP). The presence (yellow) and absence (blue) of corresponding key genes (Fig. 1) is indicated by colour.

## Data Availability

The dataset(s) supporting the conclusions of this article are all included within the article.
