# Supplementary material for: Genome-based selection and application of food-grade microbes for chickpea milk fermentation towards increased l-lysine content, elimination of indigestible sugars, and improved flavour
Source: Microb Cell Fact. 2021 May 28;20:109. doi: 10.1186/s12934-021-01595-2 (PMC8161961; doi:10.1186/s12934-021-01595-2)
Supplement: Supplementary file 1 — Additional file 1: Table S1. Growth conditions and media used to pre-culture the different strains. Table S2. Microbial growth during chickpea milk fermentation. The data are given as (colony forming units) mL-1. n=3. Table S3. The number of locus in B. amyloliquefaciens NCC 156 and L. paracasei subsp. paracasei NCC 2511 encoding functional enzymes invoving in flavor formation,pyruvate, and butanoate metabolism. Table S4. The number of locus in B. amyloliquefaciens NCC 156 and L. paracasei subsp. paracasei NCC 2511 encoding functional enzymes invoving in carbohydrate degradation and citrate metabolism. Fig. S1. Pre-treatment strategies for the processing of chickpea flour suspensions prior to microbial fermentation. Phase separation for the untreated milk (A), contamination of non-inoculated milk after pasteurization (63°C, 5 h) (B), gelation and phase separation after stronger heating (90°C, 30 min) (C), gelation and phase separation after autoclaving (121°C, 15 min), generation of homogeneous and sterile suspension after a two-step treatment that included combined heating and stirring (2 h, 75°C, 250 rpm) and autoclaving (121 °C, 15 min) (E). The sterility of pasteurized milk (A) and two-step treated milk (D), was evaluated by non-inoculated incubation for 48 h at 37°C. Fig. S2. Genomic repertoire of food-grade microbes linked to l-lysine metabolism: pathways for l-lysine biosynthesis (LSP), pathways competing with l-lysine biosynthesis for carbon precursors (PCP), and pathways for l-lysine degradation (LDP). The presence (yellow) and absence (blue) of corresponding key genes (Fig. 1) is indicated by colour. [file 12934_2021_1595_MOESM1_ESM.docx]

**Additional file to**

**Genome-based selection and application of food-grade microbes for chickpea milk fermentation towards increased l-lysine content, elimination of indigestible sugars, and improved flavour**

Muzi Tangyu^1^, Michel Fritz^1^, Rosa Aragao-Börner^2^, Lijuan Ye^2^, Biljana Bogicevic^2^, Christoph J. Bolten^3^, and Christoph Wittmann^1#^

^1^Institute of Systems Biotechnology, Saarland University, Saarbrücken, Germany

^2^Nestlé Research Center, Lausanne, Switzerland

^3^Nestlé Product Technology Center Food, Singen, Germany

Contact information

[muzi.tangyu@uni-saarland.de](mailto:muzi.tangyu@uni-saarland.de)

[m.fritz@mx.uni-saarland.de](mailto:m.fritz@mx.uni-saarland.de)

rosa.aragaoboerner@rd.nestle.com

[lijuan.ye@rd.nestle.com](mailto:lijuan.ye@rd.nestle.com)

biljana.bogicevic@rdko.nestle.com

[christophjosef.bolten@rdsi.nestle.com](mailto:christophjosef.bolten@rdsi.nestle.com)

[christoph.wittmann@uni-saarland.de](mailto:christoph.wittmann@uni-saarland.de)

^#^ Phone/FAX: +49 681 302 71970/71972, e-mail: [christoph.wittmann@uni-saarland.de](mailto:christoph.wittmann@uni-saarland.de)

**Table S1. Growth conditions and media used to pre-culture the different strains.**

| **Oxygen sensitivity** | **Strains** | **Growth medium** | **T (°C)** |
| --- | --- | --- | --- |
| **Anaerobic cultivation** | *F. sanfranciscensis* NCC 463 | mHH | 30 |
|  | *F. sanfranciscensis* NCC 2572 | mHH | 30 |
|  | *F. sanfranciscensis* NCC 2629 | mHH | 30 |
|  | *L. pontis* NCC 380 | mHH | 30 |
|  | *B. infantis* NCC 365 | MRS cystein | 37 |
|  | *B. longum* subsp*. infantis* NCC 283 | MRS cystein | 37 |
| **Aerotolerant cultivation** | *L. helveticus* NCC 1182 | MRS | 40 |
|  | *L. helveticus* NCC 1104 | MRS | 40 |
|  | *L. helveticus* NCC 158 | MRS | 40 |
|  | *L. hilgardii* NCC 1497 | MRS | 30 |
|  | *L. delbrueckii* subsp. *bulgaricus* NCC 621 | MRS | 37 |
|  | *L. plantarum* NCC 1385 | MRS | 30 |
|  | *L. brevis* NCC 372 | MRS | 30 |
|  | *L. johnsonii* NCC 2774 | MRS | 37 |
|  | *L. johnsonii* NCC 2822 | MRS | 37 |
|  | *L. johnsonii* NCC 2767 | MRS | 37 |
|  | *L. reuteri* NCC 1945 | MRS | 40 |
|  | *L. reuteri* NCC 2823 | MRS | 37 |
|  | *L. reuteri* NCC 2613 | MRS | 40 |
|  | *L. paracasei* subsp. *paracasei* NCC 2511 | MRS | 30 |
|  | *L. paracasei* NCC 2537 | MRS | 30 |
|  | *L. acidophilus* NCC 2766 | MRS | 37 |
|  | *L. jensenii* NCC 2867 (negative control) | MRS | 37 |
| **Aerobic cultivation** | *B. amyloliquefaciens* NCC 156 | BST | 40 |
|  | *B. amyloliquefaciens* NCC 2770 | BST | 40 |
|  | *B. subtilis* NCC 199 | BST | 40 |
|  | *B. licheniformis* NCC 2940 | BST | 40 |
|  | *B. flexus* NCC 2902 | BST | 40 |
|  | *B. flexus* NCC 2903 | BST | 40 |
|  | *B. pumilus* NCC 2962 | BST | 40 |
|  | *C. stationis* NCC 3013 | BHI | 30 |
|  | *C. stationis* NCC 3016 | BHI | 30 |

**Table S2. Microbial growth during chickpea milk fermentation.** The data are given as (colony forming units) mL^-1^. n=3.

| **Strains** | **0 h** | **24 h** | **48 h** |
| --- | --- | --- | --- |
| *F. sanfranciscensis* NCC 463 | 7.65 ± 0.03 | 7.08 ± 0.07 | 6.14 ± 0.14 |
| *F. sanfranciscensis* NCC 2572 | 7.63 ± 0.01 | 6.80 ± 0.02 | 6.24 ± 0.20 |
| *F. sanfranciscensis* NCC 2629 | 7.56 ± 0.10 | 6.29 ± 0.00 | 6.50 ± 0.12 |
| *L. pontis* NCC 380 | 7.72 ± 0.03 | 7.08 ± 0.09 | 7.72 ± 0.03 |
| *B. infantis* NCC 365 | 6.51 ± 0.02 | 7.07 ± 0.04 | 6.50 ± 0.11 |
| *B. longum* subsp*. infantis* NCC 283 | 6.08 ± 0.01 | 6.61 ± 0.25 | 6.71 ± 0.28 |
| *L. helveticus* NCC 1182 | 6.64 ± 0.03 | 7.02 ± 0.07 | 6.03 ± 0.02 |
| *L. helveticus* NCC 1104 | 6.40 ± 0.22 | 6.28 ± 0.12 | 5.86 ± 0.06 |
| *L. helveticus* NCC 158 | 6.64 ± 0.02 | 0.00± 0.00 | 0.00 ± 0.00 |
| *L. hilgardii* NCC 1497 | 7.50 ± 0.08 | 7.71 ± 0.08 | 7.72 ± 0.20 |
| *L. delbrueckii* subsp*. bulgaricus* NCC 621 | 7.03 ± 0.00 | 5.57 ± 0.24 | 0.00 ± 0.00 |
| *L. plantarum* NCC 1385 | 7.66 ± 0.02 | 8.68 ± 0.12 | 8.67 ± 0.06 |
| *L. brevis* NCC 372 | 7.30 ± 0.03 | 7.84 ± 0.12 | 7.82 ± 0.06 |
| *L. johnsonii* NCC 2774 | 7.32 ± 0.06 | 0.00 ± 0.00 | 0.00 ± 0.00 |
| *L. johnsonii* NCC 2822 | 7.19 ± 0.01 | 6.01 ± 0.03 | 6.04 ± 0.13 |
| *L. johnsonii* NCC 2767 | 8.47 ± 0.94 | 8.08 ± 0.41 | 6.18 ± 0.00 |
| *L. reuteri NCC* 1945 | 7.19 ± 0.04 | 8.62 ± 0.12 | 8.77 ± 0.45 |
| *L. reuteri* NCC 2823 | 5.16 ± 0.11 | 0.00 ± 0.00 | 0.00 ± 0.00 |
| *L. reuteri* NCC 2613 | 6.48 ± 0.06 | 7.91 ± 0.38 | 8.52 ± 0.44 |
| *L. paracasei* subsp*. paracasei* NCC 2511 | 7.39 ± 0.03 | 8.44 ± 0.04 | 8.48 ± 0.01 |
| *L. paracasei* NCC 2537 | 7.46 ± 0.04 | 8.51 ± 0.05 | 8.63 ± 0.14 |
| *L. acidophilus* NCC 2766 | 5.43 ± 0.01 | 0.00 ± 0.00 | 0.00 ± 0.00 |
| *L. jensenii* NCC 2867 | 7.00± 0.00 | 0.00 ± 0.00 | 0.00 ± 0.00 |
| *B. amyloliquefaciens* NCC 156 | 7.19 ± 0.12 | 8.93 ± 0.00 | 8.56 ± 0.14 |
| *B. amyloliquefaciens* NCC 2770 | 5.57 ± 0.03 | 8.77 ± 0.07 | 7.40 ± 0.00 |
| *B. subtilis* NCC 199 | 6.09 ± 0.09 | 7.89 ± 0.01 | 0.10 ± 0.00 |
| *B. licheniformis* NCC 2940 | 6.62 ± 0.08 | 8.78 ± 0.04 | 0.10 ± 0.00 |
| *B. flexus* NCC 2902 | 6.60 ± 0.11 | 9.04 ± 0.04 | 9.64 ± 0.12 |
| *B. flexus* NCC 2903 | 7.01 ± 0.05 | 9.74 ± 0.04 | 8.51 ± 0.03 |
| *B. pumilus* NCC 2962 | 7.01 ± 0.03 | 8.90 ± 0.13 | 9.02 ± 0.02 |
| *C. stationis* NCC 3013 | 7.25 ± 0.07 | 9.25 ± 0.03 | 9.32 ± 0.03 |
| *C. stationis* NCC 3016 | 6.85 ± 0.07 | 9.17 ± 0.00 | 9.33 ± 0.06 |

**Table S3. The number of locus in *B. amyloliquefaciens* NCC 156 and *L. paracasei* subsp. *paracasei* NCC 2511 encoding functional enzymes invoving in flavor formation,pyruvate, and butanoate metabolism.**

| **Function** | **Enzyme name and orthology** | **Enzyme Commision number** | ***B. amyloliquefaciens* NCC 156** | ***L. paracasei* subsp. *paracasei*** |
| --- | --- | --- | --- | --- |
| Flavor formation | Alcohol dehydrogenase (NAD) | EC 1.1.1.1 | 1 | 1 |
|  | Alcohol dehydrogenase (NADP^+^) | EC 1.1.1.2 | 3 | 1 |
|  | Aldehyde dehydrogenase | EC 1.2.1.3 | 1 | 0 |
|  | Acetaldehyde dehydrogenase | EC 1.2.1.10 | 0 | 1 |
|  | Branched-chain aminotransferase | EC 2.6.1.42 | 0 | 1 |
|  | Leucine dehydrogenase | EC 1.4.1.9 | 1 | 0 |
|  | Valine dehydrogenase (NAD^+^) | EC 1.4.1.23 | 1 | 0 |
|  | L-Amino-acid oxidase | EC 1.4.3.2 | 1 | 0 |
| Pyruvate metabolism | Lactate dehydrogenase | EC 1.1.1.27 | 2 | 2 |
|  | Pyruvate-formate lyase | EC 2.3.1.54 | 0 | 1 |
| Butanoate metabolism | Acetolactate-synthase | EC 2.2.1.6 | 3 | 1 |
|  | Acetolactate-decarboxylase | EC 4.1.1.5 | 1 | 1 |

**Table S4. The number of locus in *B. amyloliquefaciens* NCC 156 and *L. paracasei* subsp. *paracasei* NCC 2511 encoding functional enzymes invoving in carbohydrate degradation and citrate metabolism.**

| **Function** | **Enzyme name and orthology** | **Enzyme Commision number** | ***B. amyloliquefaciens* NCC 156** | ***L. paracasei* subsp. *paracasei*** |
| --- | --- | --- | --- | --- |
| Carbohydrate degradation | α-Galactosidase | EC 3.2.1.22 | 3 | 1 |
|  | β-Fructosidase | EC 3.2.1.23 | 3 | 1 |
|  | α-Amylase | EC 3.2.1.1 | 2 | 0 |
|  | Pullulanase | EC 3.2.1.41 | 1 | 0 |
|  | Neopullulanase | EC 3.2.1.135 | 1 | 1 |
| Citrate metabolism | Citrate CoA-transferase (citrate lyase α-subunit) | EC 2.8.3.10 | 0 | 1 |
|  | Citrate CoA-lyase (citrate lyase β-subunit) | EC 4.1.3.34 | 0 | 1 |
|  | Citrate lyase synthetase | EC 6.2.1.22 | 0 | 1 |


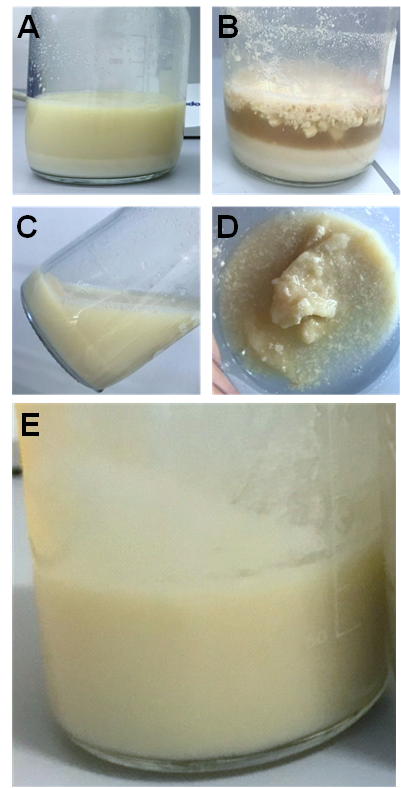


**Fig. S1: Pre-treatment strategies for the processing of chickpea flour suspensions prior to microbial fermentation.** Phase separation for the untreated milk (A), contamination of non-inoculated milk after pasteurization (63°C, 5 h) (B), gelation and phase separation after stronger heating (90°C, 30 min) (C), gelation and phase separation after autoclaving (121°C, 15 min), generation of homogeneous and sterile suspension after a two-step treatment that included combined heating and stirring (2 h, 75°C, 250 rpm) and autoclaving (121 °C, 15 min) (E). The sterility of pasteurized milk (A) and two-step treated milk (D), was evaluated by non-inoculated incubation for 48 h at 37°C.

**
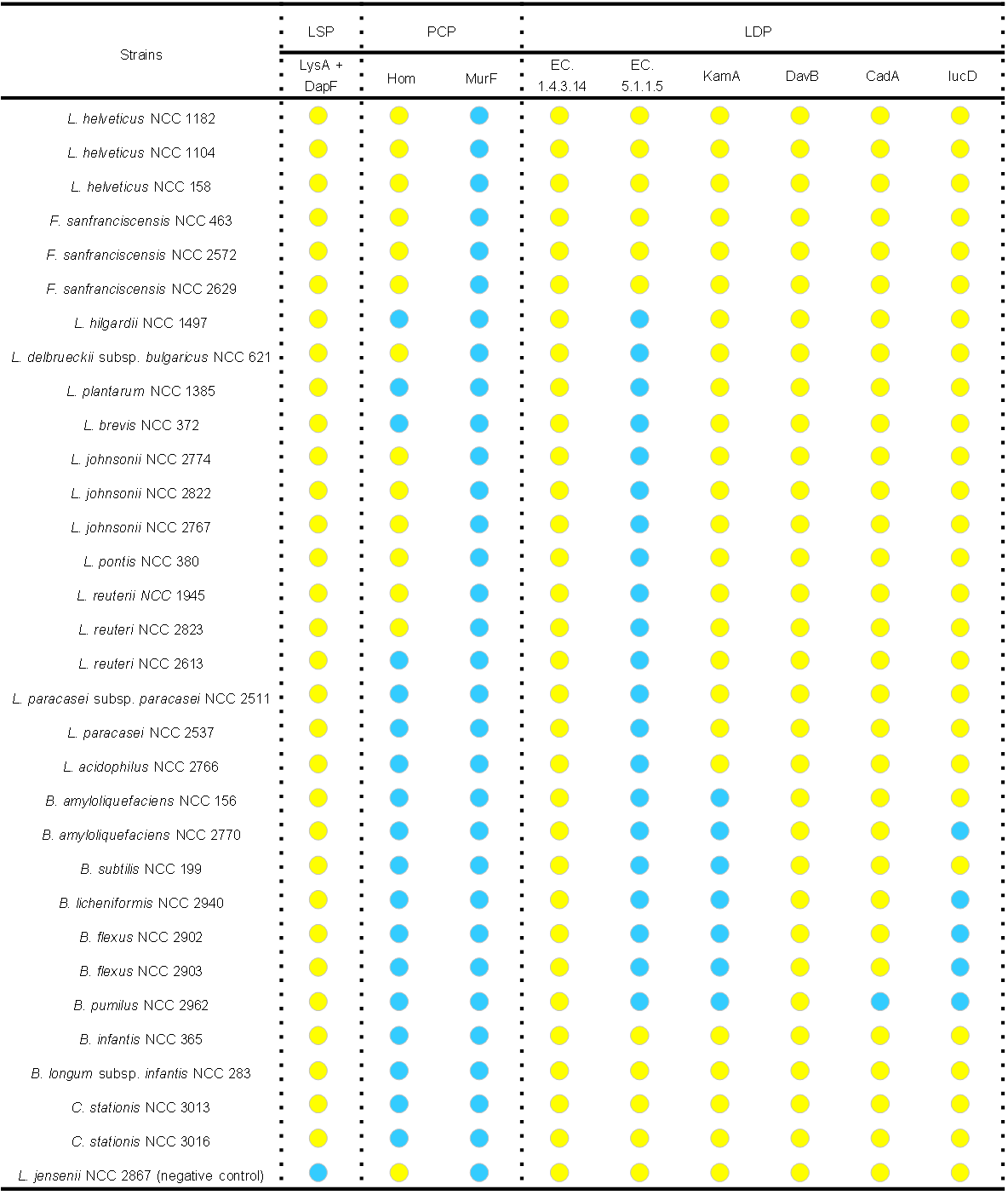
**

**Fig. S2: Genomic repertoire of food-grade microbes linked to l-lysine metabolism: pathways for l-lysine biosynthesis (LSP), pathways competing with l-lysine biosynthesis for carbon precursors (PCP), and pathways for l-lysine degradation (LDP).** The presence (yellow) and absence (blue) of corresponding key genes (**Fig. 1**) is indicated by colour.
